# Identification and Pathway Analysis of SNP Loci Affecting Abdominal Fat Deposition in Broilers

**DOI:** 10.3390/ani15192811

**Published:** 2025-09-26

**Authors:** Dachang Dou, Hengcong Chen, Yaowen Ge, Jiamei Zhou, Cheng Chang, Fuyang Zhang, Shengwei Yang, Zhiping Cao, Peng Luan, Yumao Li, Hui Zhang

**Affiliations:** College of Animal Science and Technology, Northeast Agricultural University, Harbin 150030, China; doudachang@163.com (D.D.); 15610303963@163.com (H.C.); geyaowen1998@163.com (Y.G.); zhoujiamei@neau.edu.cn (J.Z.); b230501003@neau.edu.cn (C.C.); s220501010@neau.edu.cn (F.Z.); yangshengwei@neau.edu.cn (S.Y.); caozhiping@neau.edu.cn (Z.C.); luan0901@neau.edu.cn (P.L.); liyumao@neau.edu.cn (Y.L.)

**Keywords:** broiler, abdominal fat, SNP, differentially expressed genes, pathway of action

## Abstract

**Simple Summary:**

With the accelerated growth rate of modern broilers, excessive abdominal fat deposition has emerged as a major issue negatively affecting meat quality and feed utilization efficiency. In this study, we identified over 200 genetic markers (SNPs) closely associated with abdominal fat deposition by integrating transcriptome data, whole-genome resequencing, and three-dimensional genomic information from broiler lines divergently selected for high and low abdominal fat content. These SNPs may influence fat accumulation in broilers by modulating gene expression, such as altering protein translation efficiency, mRNA structural stability, transcription factor binding capacity, or long-range genomic interactions. Our findings provide new insights into the molecular mechanisms underlying fat deposition in broilers and offer a valuable reference for molecular breeding aimed at developing leaner chicken lines.

**Abstract:**

Excessive abdominal fat deposition accompanying rapid growth in broiler chickens seriously affects production efficiency. Using divergently selected broiler lines from Northeast Agricultural University, we integrated transcriptome sequencing, whole-genome resequencing, and three-dimensional genomic data to identify key SNPs affecting abdominal fat deposition. From 3,850,758 initial SNPs, 22,721 high-quality SNPs were selected (|ΔAF| ≥ 0.9) and validated to obtain 7341 reliable SNPs. GWAS identified 16 SNPs significantly associated with abdominal fat weight, while LD analysis revealed 22 highly linked SNPs, finally determining 2302 candidate SNPs. Transcriptome analysis identified 825 differentially expressed genes (*p* ≤ 0.05, |FC| ≥ 1.5). Functional annotation revealed 201 SNPs located in differentially expressed gene regions, including 8 coding SNPs and 193 non-coding SNPs, with an additional 15 SNPs potentially regulating through long-range chromatin interactions. Mechanistic analysis showed that coding SNPs regulate gene expression by altering codon translation rates or mRNA stability, while non-coding SNPs regulate transcription by affecting transcription factor binding. Phenotypic association analysis demonstrated that all 213 SNPs can cause ≥2-fold differences in abdominal fat weight, with 182 SNPs causing ≥3-fold differences. This study successfully identified 213 functional SNPs affecting abdominal fat deposition in broilers and revealed their molecular basis for regulating fat metabolism through multiple mechanisms, providing important genetic markers for low-fat breeding in broilers.

## 1. Introduction

In recent decades, broiler breeding has primarily focused on enhancing growth performance; however, this has been accompanied by issues such as excessive abdominal fat deposition and a decline in meat quality [1]. Excessive abdominal fat not only reduces feed conversion efficiency and reproductive performance in breeder flocks but also increases the risk of obesity-related diseases and even threatens survival. Therefore, reducing abdominal fat deposition in broilers is crucial for improving both health and overall production performance.

Traditional phenotypic selection methods for broiler fat deposition have primarily relied on abdominal fat weight to distinguish between high- and low-fat lines. Early studies by Leclercq et al. [2] and Lilburn et al. [3] successfully developed lean and obese broiler lines through multi-generational selection. Later, researchers identified a correlation between plasma very low-density lipoprotein (VLDL) concentration and body fat content [4], and Whitehead et al. [5] successfully established low-fat broiler lines using plasma VLDL concentration as the selection criterion. However, these traditional approaches are limited by inherent drawbacks, including long breeding cycles, susceptibility to errors, and high labor costs.

With the advancement of molecular genetics and genomics, broiler breeding has gradually shifted from traditional approaches to molecular breeding. Molecular marker-assisted selection (MAS) enables precision breeding by integrating molecular markers with phenotypic and genotypic information. For instance, Sun et al. [6] reported that multiple SNPs within the PPARG gene were significantly associated with breast muscle intramuscular fat content and abdominal fat weight, providing a genetic basis for MAS. Broiler body fat traits are complex quantitative traits regulated by numerous polygenes with small effects [7]. Recent studies have demonstrated that single nucleotide polymorphisms (SNPs) can alter gene expression and thereby influence fat deposition. For example, Zhang et al. [8] identified a SNP (rs314613110) in the AMY1A gene that affects the binding of miRNA gga-miR-1764-3p to the 3′UTR, leading to altered gene expression and changes in leg muscle and abdominal fat deposition. Similarly, Cheng et al. [9] found 11 SNPs within intron 17 of the RB1 gene significantly associated with abdominal fat weight and fat percentage. Further analysis revealed that the A allele of one SNP (g.32828A>G) inhibited RB1 expression by binding to NF-B-K and SOX2, thereby promoting abdominal fat deposition. In addition, Trevisoli et al. [10] applied additive modeling to predict deleterious SNPs and identified a mutation (c.482C>T) in exon 2 of the MYBPH gene that was significantly associated with abdominal fat weight and percentage, suggesting that this variant may contribute to fat accumulation in broilers through altered gene expression.

Single nucleotide polymorphisms (SNPs) are the most common type of genetic variation and are widely distributed across the broiler genome, including the 5′ and 3′ untranslated regions (UTRs), introns, exons, and intergenic regions. Previous studies have suggested that nonsynonymous mutations in exonic regions are more likely to exert functional effects than other variants, as they can alter amino acid sequences and thereby influence protein structure and folding [11]. In contrast, synonymous mutations have traditionally been overlooked because they do not change the amino acid sequence. However, increasing evidence indicates that both synonymous and nonsynonymous mutations can affect gene expression [12,13,14]. For instance, synonymous mutations may influence translation efficiency and the folding of mRNA secondary structures [15]. Moreover, SNPs in non-coding regions are more abundant than those in coding regions [16]. Such variants can regulate gene expression by disrupting splice sites, modifying transcription factor binding, or altering the activity of distant regulatory elements. Collectively, SNPs located in different genomic regions can regulate gene expression through diverse mechanisms, ultimately influencing phenotypic traits [17].

SNPs, as important genetic markers, play a crucial role in broiler molecular breeding. Advances in detection technologies and functional studies have provided a solid theoretical foundation for understanding the regulatory roles of SNPs in economically important traits, thereby promoting the development of molecular breeding strategies. However, abdominal fat deposition is a complex quantitative trait, and current identified SNPs are insufficient to fully explain the genetic basis underlying this trait variation. Therefore, the present study aimed to identify differentially expressed genes and SNPs between high- and low-fat broiler lines using transcriptomic data, and to further investigate the regulatory pathways of SNPs involved in abdominal fat deposition by integrating genome resequencing and three-dimensional genomic data.

## 2. Materials and Methods

### 2.1. Materials

The experimental birds used in this study were the high-fat line (FL) and low-fat line (LL) broilers developed at Northeast Agricultural University. These lines are the only fast-growing broiler bi-directional selection lines in China. They were established by our research group in 1996 using Arbor Acres (AA) broilers as the founder population and subjected to strict bi-directional selection for abdominal fat content. After continuous generations of selection, the FL and LL lines have become highly divergent in fat-related traits such as abdominal fat weight and abdominal fat percentage, exhibiting significant differences [18]. All birds were raised under identical environmental conditions with ad libitum access to feed and water. The room temperature was maintained at 18–25 °C with a relative humidity of 60–65%. The diets were formulated according to NRC (1994) standards [19]. From hatching to 3 weeks of age, birds were fed a starter diet [metabolizable energy (ME), 3000 kcal/kg; crude protein (CP), 210 g/kg]. From 4 weeks of age until 7 weeks, a grower diet was provided (ME, 3100 kcal/kg; CP, 190 g/kg). At 7 weeks of age, all experimental birds were handled in accordance with the Animal Welfare and Ethics Requirements of Northeast Agricultural University. After slaughter, abdominal adipose tissue was separated, weighed, rinsed with 0.75% NaCl solution, rapidly frozen in liquid nitrogen, and stored at −80 °C until further use.

Genomic resequencing data were generated from abdominal adipose tissues of 7-week-old broilers, including 160 birds from the FL and 170 birds from the LL, for a total of 330 individuals. Transcriptome data were obtained from RNA-seq of abdominal adipose tissues from 7-week-old FL and LL broilers (five birds per line). Three-dimensional genomic data were obtained from Hi-C sequencing of abdominal adipose tissues from 7-week-old FL and LL broilers (three birds per line).

### 2.2. Methods

#### 2.2.1. RNA-Seq-Based SNP Detection and Quality Control

Raw transcriptome data were subjected to quality control using Fastp(v1.0.1) [20] to remove adapters and low-quality reads, thereby generating high-quality clean reads. The clean reads were then aligned to the *Gallus gallus* reference genome (GRCg6a, Ensembl) using HISAT2(v2.2.1) [21,22,23]. SNP detection was performed with the GATK(v4.6.1.0) software package following the standard pipeline. Briefly, duplicate reads were removed using the MarkDuplicates(v4.0.1.1) tool, and HaplotypeCaller(v4.1.7.0) was applied to reconstruct haplotypes for each sample and generate individual gVCF files. These gVCF files were subsequently merged with CombineGVCFs to produce a single dataset containing all SNP information, followed by joint genotyping with GenotypeGVCFs to obtain a multi-sample VCF file. Low-quality variants were filtered using VariantFiltration(v4.1.6.0) with the thresholds FS > 30.0 and QD < 2.0, resulting in a high-confidence SNP set.

Allele frequency differences between high- and low-fat broiler lines were calculated using the –freq parameter in VCFtools(v0.1.16). Differential SNPs were preliminarily screened based on the absolute allele frequency difference (|ΔAF|) between the two lines, with a threshold of |ΔAF| ≥ 0.9.

#### 2.2.2. Screening SNPs with Combined Genome Resequencing Data

Following the preliminary screening of differential SNPs based on allele frequency differences between high- and low-fat lines, genome resequencing data from 330 samples were used to further eliminate false positives and low-quality variants, thereby retaining only high-confidence SNPs for subsequent analyses. Differences in allele frequencies and chi-square values between the two lines were calculated using PLINK(v1.9) and Popgene (v1.32) to identify SNPs potentially associated with abdominal fat deposition.

**Figure 1:**
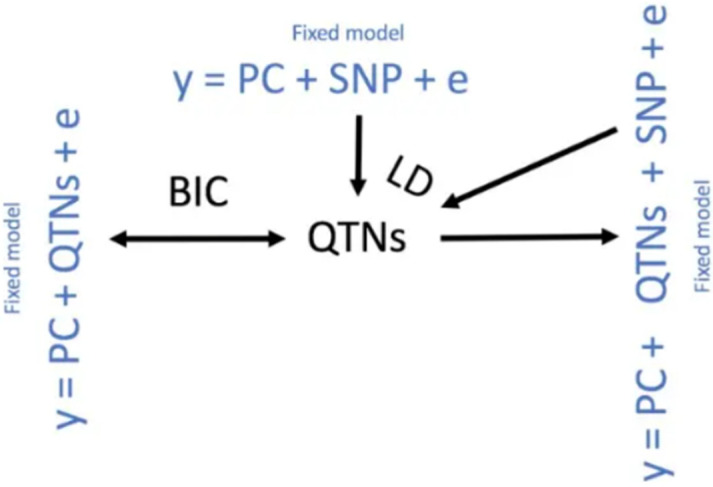
Blink model schematic.

Genome-wide association analysis (GWAS) was then conducted on the high-quality SNPs using the BLINK model implemented in GAPIT [24], with a stringent Bonferroni correction applied to identify SNPs significantly associated with abdominal fat traits. Briefly, the BLINK model detects quantitative trait nucleotides (QTNs) through an iterative process: (i) candidate QTNs are first identified with the model y = PC + SNP + e; (ii) these QTNs are then included as covariates in the model y = PC + QTNs + SNP + e to test additional SNPs; (iii) linkage disequilibrium (LD) information is used to refine QTN detection; and (iv) the Bayesian Information Criterion (BIC) is applied with the model y = PC + QTNs + e to exclude false positives while retaining true QTNs. This cycle is repeated until all QTNs are identified (Figure 1). Finally, SNPs significantly associated with abdominal fat weight were subjected to LD analysis using LDBlockShow(v1.41) to characterize their genomic distribution.

#### 2.2.3. Screening for SNPs Potentially Affecting the Expression of Genes Related to Abdominal Fat Deposition

To identify SNPs that may influence the expression of genes associated with abdominal fat deposition, transcriptome RNA-seq data were analyzed. Transcript assembly and quantification of expressed genes were performed using StringTie(2.2.3), and differential expression analysis was conducted with Ballgown in R(4.2.0) [25]. Differentially expressed genes (DEGs) between high-fat and low-fat broilers were identified using a significance threshold of *p* ≤ 0.05 and a fold-change (FC) >1.5 or <0.67.

Based on these DEGs and integrated three-dimensional genomic (Hi-C) data, candidate SNPs were categorized into three groups: (i) SNPs located in coding regions of DEGs, (ii) SNPs located in non-coding regions of DEGs, and (iii) SNPs located outside DEGs but potentially exerting distal regulatory effects on DEGs through chromatin interactions. These SNPs were considered as candidate variants for subsequent analyses.

#### 2.2.4. Prediction of SNP-Regulated Gene Expression Pathways

Based on the candidate SNPs, their potential effects on the expression of genes related to abdominal fat deposition in broilers were further investigated using multiple bioinformatics approaches. For SNPs located in coding regions of differentially expressed genes, six tools—including NetPhos(v3.1) [26,27], NetNGlyc(v1.0) [28], NovoPro (www.novoprolabs.com, accessed on 21 September 2025), SOPMA (npsa.lyon.inserm.fr/cgi-bin, accessed on 21 September 2025), ExpOptimizer (https://www.novoprolabs.com/tools/codon-optimization, accessed on 21 September 2025), and RNAfold (http://rna.tbi.univie.ac.at/cgi-bin/RNAWebSuite/RNAfold.cgi, accessed on 21 September 2025)—were employed to assess potential impacts on phosphorylation and glycosylation modifications, protein hydrophobicity, secondary structure, codon translation efficiency, and mRNA secondary structure.

For SNPs located in non-coding regions of differentially expressed genes, AnimalTFDB(v4.0) and TFBIND(v3.4) [29] were used to evaluate whether the mutations altered transcription factor binding sites or their affinities. For SNPs potentially regulating distal gene expression, AnimalTFDB, TFBIND, and IGV were applied to examine changes in transcription factor binding and inter-genomic interactions. The results of these analyses were integrated to identify SNPs most likely to regulate abdominal fat deposition in broiler chickens.

### 2.3. Statistical and Bioinformatics Analysis

#### 2.3.1. Principal Component Analysis

Principal component analysis (PCA) was performed using the 7341 validated SNPs identified through the integration of transcriptome and genome resequencing data to assess population structure and genetic relationships between high-fat (FL) and low-fat (LL) broiler lines. PCA was implemented using PLINK v1.9 software, and the first two principal components were visualized to demonstrate the clear separation between the two divergent lines.

#### 2.3.2. Additional Statistical Methods

Multiple statistical approaches were employed to ensure the accuracy and reliability of the analytical results. For GWAS, the Bonferroni method was applied for multiple testing correction to control the false discovery rate. Chi-square tests were used to analyze allele frequency differences between high-fat and low-fat broiler lines, assessing the degree of population differentiation at SNP loci. For comparison of continuous variables between groups, appropriate statistical tests were selected based on data distribution characteristics: Student’s *t*-tests were applied for normally distributed data, while Mann–Whitney U tests were used for non-normally distributed data. Additionally, hierarchical clustering analysis of gene expression patterns was performed using the R(v4.2.0) package pheatmap to identify gene clusters with similar expression profiles and validate differential expression patterns between high-fat and low-fat broiler lines.

## 3. Results

### 3.1. Screening Differential SNPs Between High- and Low-Fat Lines in Abdominal Adipose Tissue Based on Transcriptomic Data

**Figure 2:**
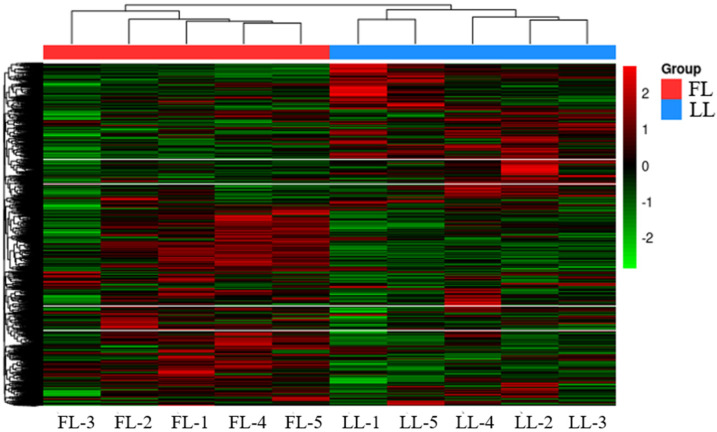
Gene expression clusterin. Note: FL represents high−fat individuals, and LL represents low-fat individuals.

In this study, raw sequencing data were first subjected to quality control using Fastp. After removing low-quality reads, reads containing ambiguous bases (N), and overly short reads, high-quality data were obtained from 10 libraries. On average, the high- and low-fat line libraries yielded 87,673,925 and 83,745,064 clean reads, respectively, with Q20 values exceeding 95% for all samples. The clean reads were then aligned to the chicken reference genome (GRCg6a) using Hisat2(v2.2.1), achieving mapping rates of 89.97–91.18%, which confirmed the reliability of the sequencing data for subsequent analyses. Furthermore, hierarchical clustering of gene expression using the R package pheatmap (Figure 2) revealed distinct gene expression patterns between high- and low-fat lines, validating the usability of the sequencing dataset.

SNP calling was performed using GATK(v4.6.1.0) on the aligned data. To reduce potential false positives, stringent filtering thresholds were applied (FS > 30.0, QD < 2.0). After filtering, a total of 3,850,758 SNPs were identified. Subsequently, allele frequencies of each SNP were calculated separately for the high- and low-fat lines, and SNPs with an absolute allele frequency difference (|ΔAF|) ≥ 0.9 were retained for further analysis. This process yielded a final set of 22,721 SNPs (Table 1).

### 3.2. Identification of SNPs Associated with Abdominal Fat Deposition in Broilers Based on Genome Resequencing Data

**Figure 3:**
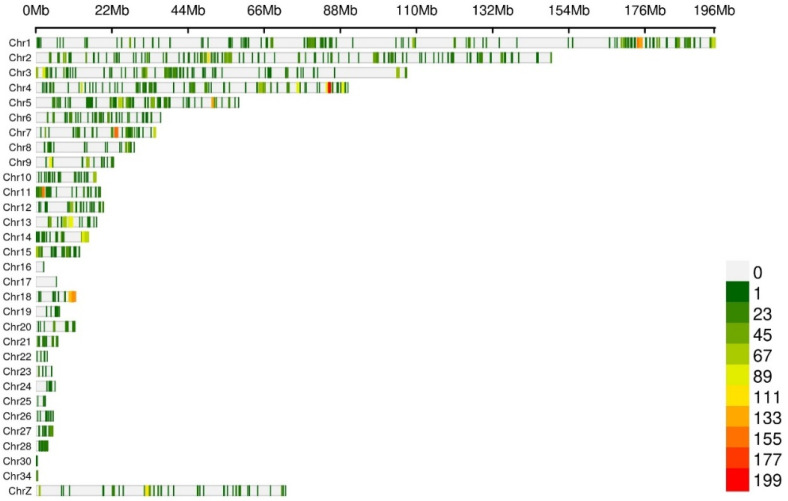
SNP density distribution map.

To improve the accuracy of SNP locus identification, validation was performed using resequencing data. Specifically, the 22,721 differential SNPs previously identified from transcriptome data were cross-validated against existing resequencing data comprising 330 samples from the study population. Among these, 7341 SNP loci were consistently detected in both datasets. These validated loci were therefore selected as the focus of subsequent analyses (Figure 3).

#### 3.2.1. Allele Frequency Analysis

**Figure 4:**
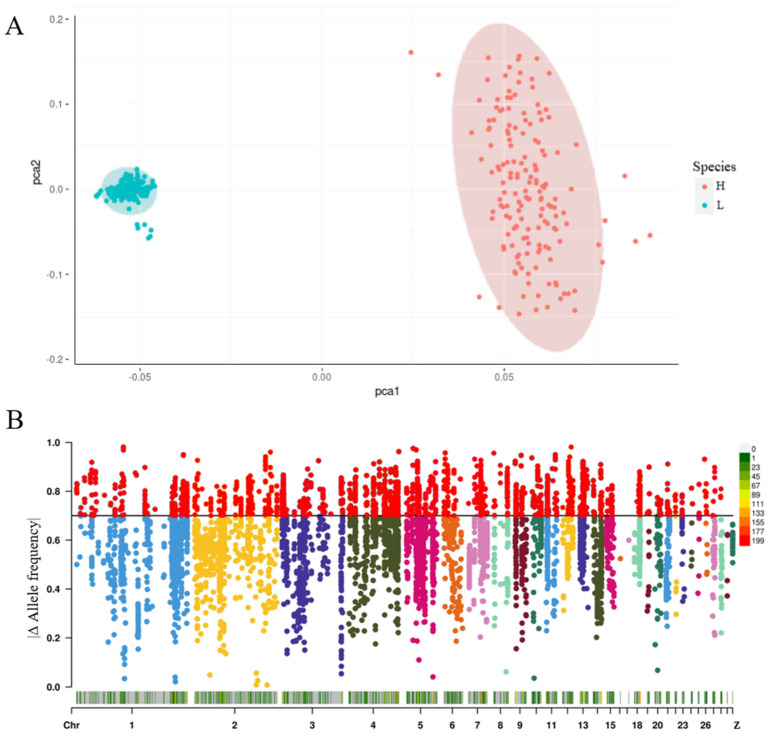
Principal component analysis map and allele frequency difference map. (**A**) Principal Component Analysis (PCA) plot showing the genetic structure differentiation between two species (H and L). Cyan points represent species H, and red points represent species L. The two species show clear separation along the first principal component axis (PCA1), indicating significant genetic differentiation. (**B**) Genome-wide allele frequency difference plot (Δ Allele frequency) displaying the allele frequency differences in SNP loci across chromosomes between the two species. The Y−axis represents allele frequency difference values (0–1), and the X−axis represents chromosomal positions (Chr 1-Z), with different colors indicating different chromosomes.

In this experiment, the 7341 SNPs jointly identified by transcriptome and resequencing data were subjected to principal component analysis (PCA). The results indicated that the 330 samples were clearly separated into two relatively independent clusters (Figure 4A). Subsequently, we calculated the allele frequencies of these SNPs in the high- and low-lipid lines. A total of 2281 SNPs with significant frequency differences (|Δ allele frequency| ≥ 0.7) were identified (Figure 4B).

#### 3.2.2. Genome-Wide Association Analysis (GWAS) of Abdominal Fat Weight

**Figure 5:**
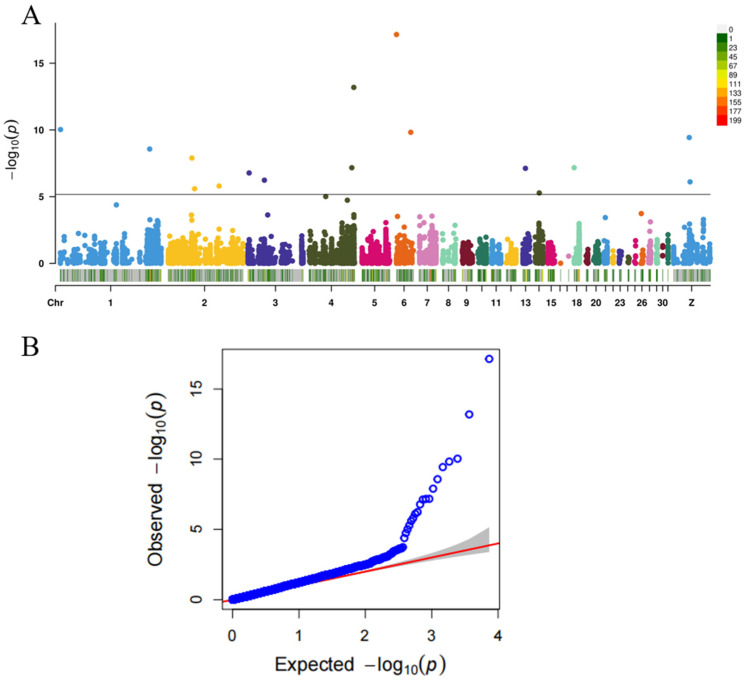
Genome-wide association analysis of abdominal fat weight. (**A**) shows the Manhattan chart, the black line indicates the threshold for screening SNP, and the bottom of the Manhattan chart shows the SNP density distribution. (**B**) is a QMQ diagram.

In addition to allele frequency analysis, this study employed the Blink model to examine the association between the 7341 SNPs identified through resequencing and transcriptome integration and abdominal fat weight. The GWAS results revealed that 16 SNPs were significantly associated with abdominal fat weight at the genome-wide significance threshold determined by Bonferroni correction (−log10(P) > 5.16), indicating their strong correlation with this trait (Figure 5).

#### 3.2.3. Linkage Disequilibrium (LD) Analysis

**Figure 6:**
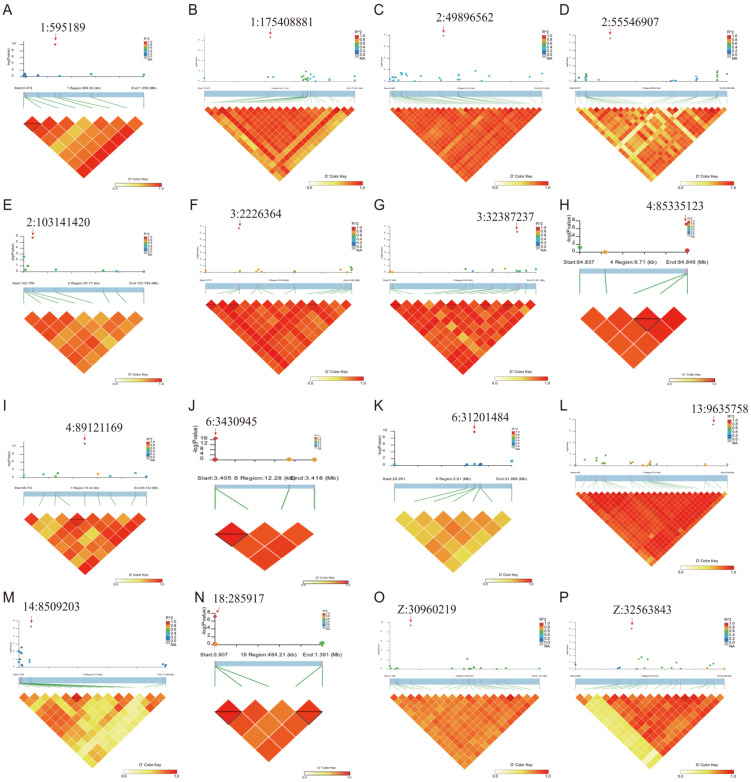
Linkage disequilibrium analysis diagram. Panels (**A**–**P**) correspond to the top SNPs identified by GWAS on different chromosomes. In each subfigure, the upper part shows the regional Manhattan plot, where the lead SNP is indicated by a red arrow. The lower part depicts the pairwise LD structure (*r*^2^) among SNPs in the region, with color intensity from yellow to red representing low to high LD. Specifically, subfigures A–P illustrate representative loci on chromosomes 1, 2, 3, 4, 6, 13, 14, and Z. Together, these plots highlight the genomic regions most strongly associated with abdominal fat deposition.

To further investigate the SNPs associated with abdominal fat weight, we conducted linkage disequilibrium (LD) analysis on the 16 SNPs identified by GWAS using LDBlockShow (v1.41). The results revealed that 8 of these SNPs were in strong LD with neighboring variants (Figure 6B,F–J,L,N). However, despite their strong linkage, these SNPs did not reach genome-wide significance in the association analysis.

### 3.3. Identification of Target Genes Regulated by SNPs Associated with Abdominal Fat Deposition in Broilers

#### 3.3.1. Identification of Differentially Expressed Genes Between Abdominal Adipose Tissues of Broilers from High- and Low-Fat Lines

**Figure 7:**
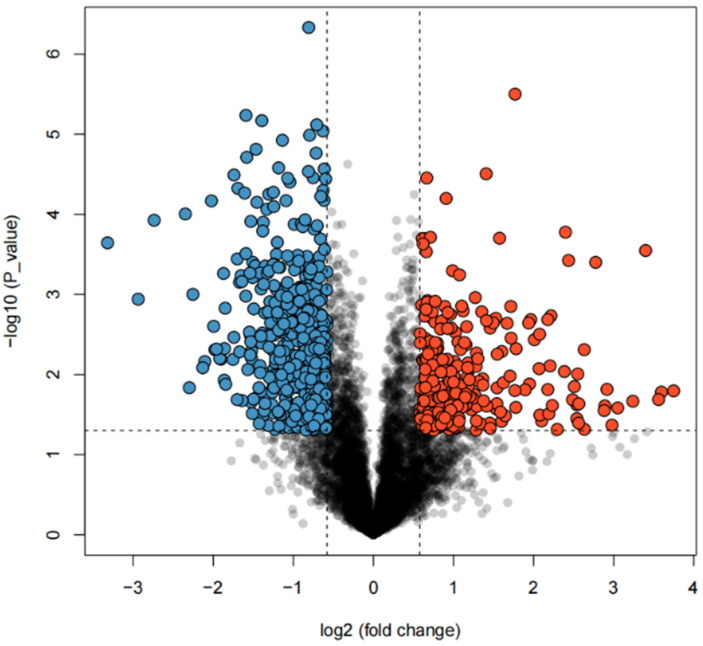
Volcanic map of differentially expressed genes. Volcano plot showing the results of differential gene expression analysis. The *X*-axis represents the log2 fold change in gene expression, and the *Y*-axis represents the negative log10 P value indicating statistical significance. Blue dots represent significantly downregulated genes (log2 fold change < −1, *p* < 0.05), red dots represent significantly upregulated genes (log2 fold change > 1, *p* < 0.05), and gray dots represent genes with no significant difference. The horizontal dashed line indicates the significance threshold (*p* = 0.05), and the vertical dashed lines indicate the fold change threshold (|log2 fold change| = 1).

To investigate the target genes potentially regulated by SNPs associated with abdominal fat deposition in broilers, transcriptome data from abdominal adipose tissues of high- and low-fat line broilers were analyzed in this study. Gene expression abundance was first estimated using Stringtie (2.2.3), and the R package Ballgown was employed to identify differentially expressed s genes between the two lines. A total of 6475 differentially expressed genes were initially identified. Applying more stringent criteria (*p* ≤ 0.05 and fold change >1.5 or <0.67), 825 genes were found to be significantly differentially expressed between high- and low-fat lines (Figure 7). Further analysis showed that, relative to the high-fat line, 286 genes were up-regulated and 539 genes were down-regulated in the low-fat line.

#### 3.3.2. SNPs Affect Broiler Abdominal Fat Deposition by Regulating Their Own Gene Expression

**Figure 8:**
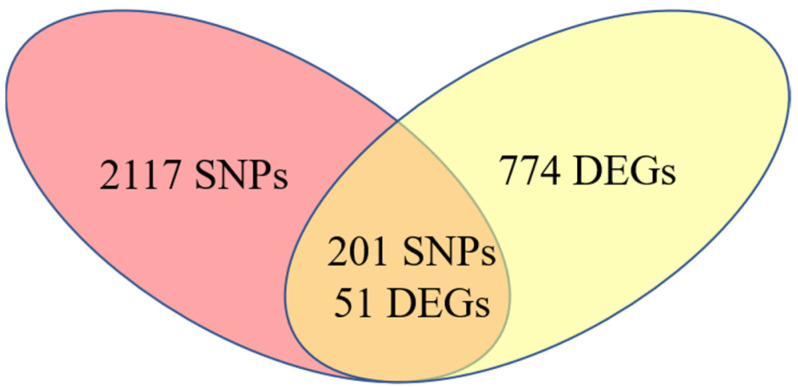
Schematic diagram of the number of SNP located in differentially expressed genes. The red region represents the number of differentially expressed genes in the 2302 SNPs; yellow regions analyzed by GWAS and LD, and the overlapping region represents the number of 201 differentially expressed genes.

To investigate the relationship between SNPs associated with broiler abdominal fat deposition and gene expression, Bedtools software (v2.31.1) was used to integrate and analyze the 2302 SNPs and 825 differentially expressed genes obtained from the above analyses. The results revealed that 201 SNPs were located within 51 differentially expressed genes (Figure 8). Among these, 2 SNPs were located in upstream regions, 1 in the 5′ UTR, 186 in intronic regions, 8 in exonic regions, 3 in the 3′ UTR, and 1 in a downstream region (Supplementary Materials Table S1). It is worth noting that during the identification of differentially expressed genes, a small number of SNPs were annotated to intergenic regions because the software automatically assigned names to unannotated genes; these SNPs were discarded during the annotation process.

#### 3.3.3. SNPs Affect Broiler Abdominal Fat Deposition Through Long-Distance Regulation of Target Gene Expression

Three-dimensional genome analysis can reveal the physical interactions and spatial organization between different genomic regions, providing insights into gene regulatory mechanisms. To investigate whether the 2302 SNPs identified above could influence abdominal fat deposition in broilers by regulating target genes at a distance, these SNPs were further analyzed in combination with the three-dimensional genome (Hi-C) data previously generated by our research group. The analysis revealed that 15 SNPs could regulate the expression of distal target genes by altering the 3D genomic structure (Table 2).

### 3.4. Bioinformatics Analysis of the Pathways by Which SNPs Regulate Target Gene Expression

#### 3.4.1. SNPs Regulate Their Own Gene Expression by Altering Protein Expression

SNPs can influence the epigenetic modification of proteins and thereby regulate the expression of target genes. Further analysis revealed that, among the 201 SNPs located in differentially expressed genes, eight SNPs (rs313638395, rs315605586, rs15674853, rs316805216, rs314812968, rs15679530, rs734759241, and rs316935384) are located in the exonic regions of seven genes, including LAMA3, PAK3, STBD1, SYT15, MYO1E, NHP2, and ENGASE, with both rs316805216 and rs314812968 located in SYT15. We hypothesize that these SNPs may affect target gene expression by altering codon translation rates, mRNA secondary structure, and other related mechanisms.

##### Codon Translation Rate Analysis

The online tool ExpOptimizer (https://www.novoprolabs.com/tools/codon-optimization, accessed on 21 September 2025) was employed to evaluate the impact of mutations on codon translation rates. The analysis showed that the rs315605586 mutation accelerates the codon translation rate, whereas the rs15674853 and rs314812968 mutations reduce it (Table 3).

##### Analysis of mRNA Secondary Structure

**Figure 9:**
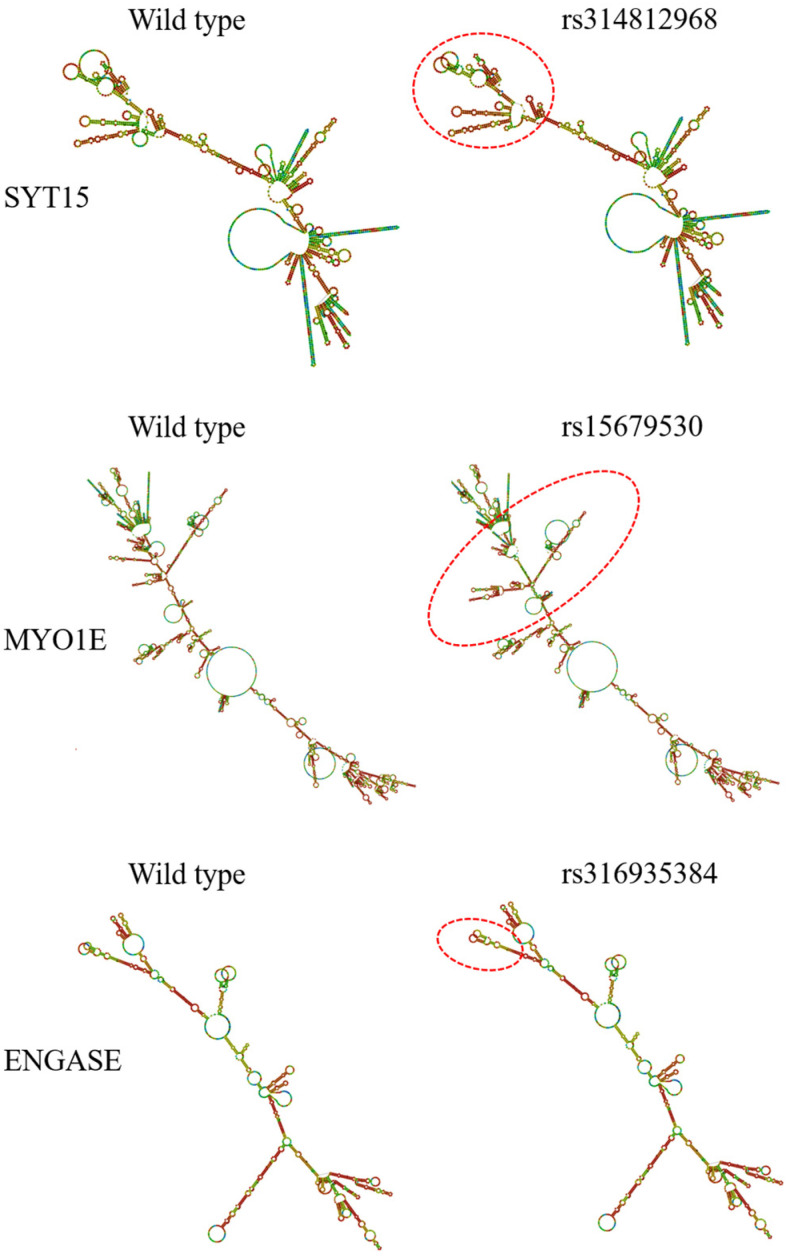
mRNA secondary structure analysis diagram. Predicted secondary structures of mRNA for wild type and variant forms of SYT15 (rs314812968), MYO1E (rs15679530), and ENGASE (rs316935384) genes. Red circles indicate regions where structural changes occur due to the genetic variants.

Analysis of mRNA secondary structure using the RNAfold (rna.tbi.univie.ac.at/cgi-bin/RNAWebSuite/RNAfold.cgi, accessed on 21 September 2025) tool revealed that three SNPs—rs314812968, rs15679530, and rs316935384—alter mRNA secondary structure. Specifically, the rs314812968 and rs316935384 mutations reduce the free energy of the mRNA secondary structure, with rs314812968 decreasing it from −368.47 kcal/mol to −371.30 kcal/mol and rs316935384 from −589.77 kcal/mol to −597.97 kcal/mol. In contrast, the rs15679530 mutation increases the free energy from −939.70 kcal/mol to −918.68 kcal/mol. These results indicate that the rs314812968 and rs316935384 mutations enhance the stability of mRNA secondary structure, whereas the rs15679530 mutation decreases its stability (Figure 9).

#### 3.4.2. SNPs Regulate Their Own Gene Expression by Modulating Transcription Factor Binding Site Activity

A total of 193 SNPs located in non-coding regions of differentially expressed genes were analyzed. The results showed that 192 of these SNPs exhibited changes in transcription factor binding sites before and after mutation, as predicted by Animal TFDB (Supplementary Materials Table S2). Furthermore, TFBIND analysis indicated that all 193 SNPs caused alterations in transcription factor binding affinities (Supplementary Materials Table S3).

#### 3.4.3. SNPs Regulate Distal Gene Expression by Modulating Transcription Factor Binding Site Activity

**Figure 10:**
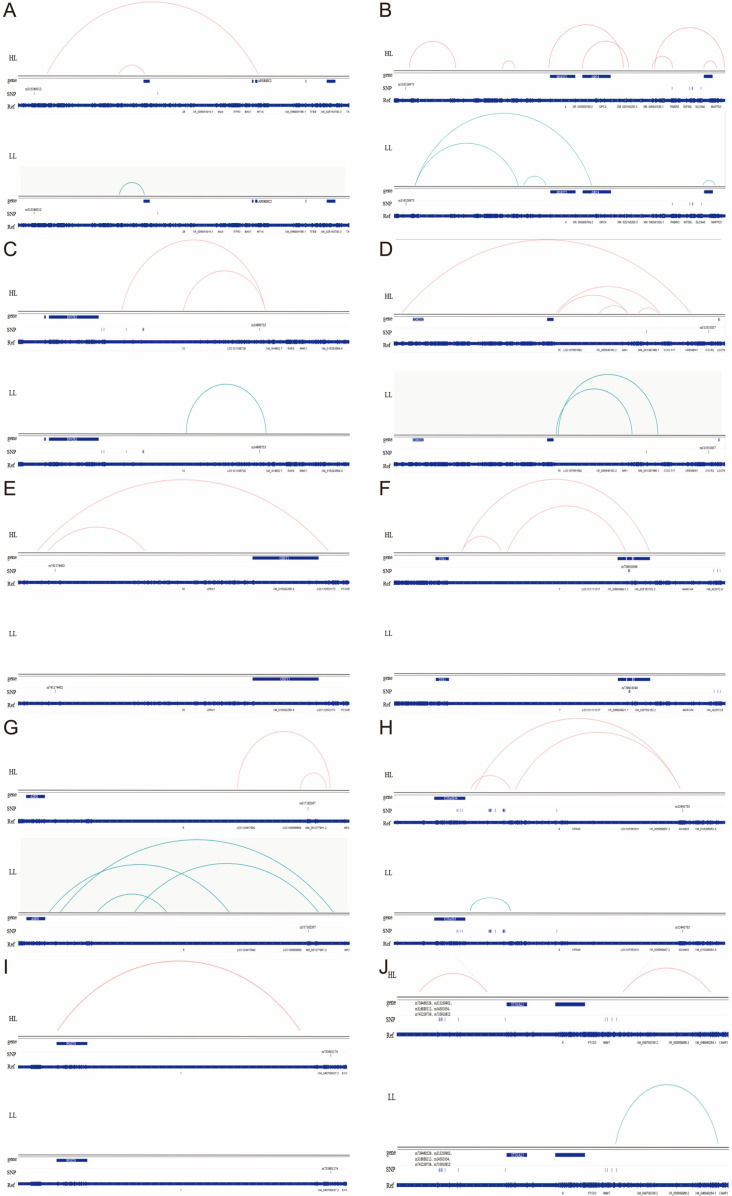
Interaction map between SNP and differentially expressed genes. (**A**–**J**) Individual interaction profiles for each of the 15 analyzed SNPs, displaying three-dimensional chromatin looping patterns between variant loci and target genes. Each panel shows the genomic region containing the SNP (blue bars represent genes/regulatory elements) and curved arcs indicating long-range chromatin interactions.

Fifteen SNPs located outside differentially expressed genes, which may remotely regulate the expression of these genes, were analyzed using AnimalTFDB(v4.0)and TFBIND (v3.4). The results revealed that these 15 SNPs induced changes in both transcription factor binding sites and binding affinities before and after mutation (Table 4 and Table 5). These changes may facilitate the regulation of distal gene expression by altering genomic interaction regions, thereby affecting distant enhancers or promoters (Figure 10).

### 3.5. Validation of the Effects of SNPs on Phenotype

**Figure 11:**
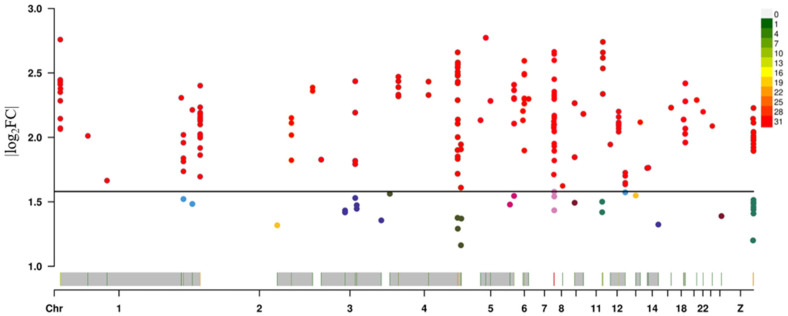
SNP affects differences in abdominal fat weight. The SNPs above the black line represent those that lead to a threefold or greater difference in abdominal fat weight.

The 213 SNPs associated with abdominal fat deposition in broiler chickens, identified from the analyses above, were further examined in combination with phenotypic data to validate their actual impact on the trait. The results showed that all 213 SNPs could induce more than a 2-fold change in abdominal fat weight, among which 182 SNPs could cause more than a 3-fold change (Figure 11). These findings confirm that the effects of these SNPs on abdominal fat deposition are genuine.

## 4. Discussion

### 4.1. Identification of SNP Loci Associated with Abdominal Fat Deposition in Broiler Chickens by Integrating Transcriptomic and Genomic Data

In this study, abdominal adipose tissues from broilers of high-fat and low-fat selection lines were used as experimental materials. Transcriptomic data obtained by RNA-seq were employed to identify SNP loci across the whole genome. A total of 3,850,758 SNPs were detected. Given that the two selection lines had undergone long-term divergent selection for abdominal fat and exhibited nearly a 10-fold difference in abdominal fat percentage, we hypothesized that SNP markers with allele frequency shifts between the lines may contribute to abdominal fat deposition. Therefore, allele frequency differences between the high- and low-fat lines were analyzed, resulting in the identification of 22,721 SNPs potentially associated with abdominal fat deposition. The rationale for first using transcriptome data rather than genome resequencing to screen SNPs was that transcriptome-derived SNPs can be directly linked to gene expression, thereby providing insights into their potential effects on phenotype. Jehl et al. [30] also demonstrated that SNP detection using GATK(v4.6.1.0) on chicken transcriptome data is feasible and can be applied to studies of gene expression and regulation across different tissues in humans. However, due to the relatively small sample size of transcriptome sequencing in this study, we anticipated a substantial number of false positives. To address this limitation, we further validated the identified SNPs using resequencing data from a large population of 330 broilers (160 from the low-fat line and 170 from the high-fat line). Among the 22,721 SNPs, only 7341 SNPs were confirmed in the resequencing dataset, supporting our suspicion of false positives in the transcriptome-only analysis. Thus, the 7341 SNPs identified through the combined transcriptomic and genomic resequencing approach are considered to be more reliable and were retained for subsequent analyses. The integration of transcriptomic and genomic approaches has been effectively applied in various livestock species to identify genetic variants associated with economically important traits. For example, Yang et al. [31] combined RNA-seq and whole-genome sequencing data to pinpoint SNPs influencing intramuscular fat content in pigs, while Jiang et al. [32] employed a similar strategy to investigate biological processes related to rumen function and lipid metabolism. These studies consistently show that joint analyses substantially reduce false-positive rates compared with single-platform approaches, providing strong support for our methodological framework.

Next, using genome resequencing data from 330 individuals of broilers from high- and low-fat lines, we further analyzed allele frequency differences in the 7341 candidate SNPs. The results showed that 2281 SNPs exhibited significant allele frequency differences between the two lines, suggesting that these SNPs may serve as important genetic markers influencing abdominal fat deposition. In parallel, we performed a GWAS to assess the association between the 7341 SNP polymorphisms and abdominal fat weight. This analysis identified 16 SNPs that were significantly correlated with abdominal fat weight. Subsequent linkage disequilibrium (LD) analysis revealed that an additional 22 SNPs in the surrounding genomic regions were in strong LD with these 16 SNPs. Altogether, 38 SNPs identified through GWAS and LD analysis were mapped to 16 genes, supporting the notion that abdominal fat deposition is a complex trait regulated by multiple genetic factors. Functional annotation of the 16 genes revealed that LAMA3, SH3PXD2B, and GAS7 are closely associated with adipogenesis. Specifically, LAMA3 has been shown to participate in adaptogenic differentiation, with its expression significantly downregulated during this process [33]. SH3PXD2B is implicated in lipid biosynthesis [34] and plays a role in early growth in Large White pigs [35]. Moreover, mutations in GAS7 have been reported to increase body fat levels and reduce bone mineral density in mice [36]. Taken together, these findings not only validate the reliability of our integrative approach but also suggest that the identified SNPs and their associated genes may play important roles in regulating abdominal fat deposition in broilers.

### 4.2. Identification of Target Genes for SNP Regulation Related to Abdominal Fat Deposition in Broiler Chickens

Combining the results of allele frequency analysis, GWAS, and LD analysis, a total of 2302 SNP markers potentially associated with abdominal fat deposition in broilers were identified. These markers may play a role in fat deposition; however, it remains unclear whether their effects on the trait are mediated through changes in gene expression. To address this question, we further utilized transcriptomic data from abdominal adipose tissues of broilers from the high- and low-fat lines to perform differential gene expression analysis, aiming to identify genes with significant expression changes related to abdominal fat deposition and to elucidate their potential regulatory roles. As a result, 825 differentially expressed genes (DEGs) were identified between the two lines. Compared with the high-fat line, 286 genes were significantly upregulated and 539 genes were significantly downregulated in the low-fat line. The criteria used for DEG screening in this study were consistent with those applied in previous studies, namely *p* ≤ 0.05 and fold change (FC) >1.5 or <0.67. For instance, Wang et al. [37] adopted the same criteria to identify DEGs in chicken adipose tissue, while Wu et al. [38] applied them to detect 191 differentially expressed miRNAs between liver tissues of neonatal and adult chickens. These examples demonstrate the reliability and appropriateness of the screening criteria employed in this study.

Combining the above screened 2302 SNPs that may be associated with abdominal fat deposition in broilers with 825 differentially expressed genes (DEGs) in abdominal adipose tissue of high- and low-fat line broilers, 201 SNPs were found to be located on 51 DEGs. Among these 201 SNPs, only 8 were located in the exonic regions, while the remaining SNPs were in non-coding regions, which may be explained by the fact that non-coding regions account for a large proportion of the genome and therefore harbor more mutation sites [39,40,41]. Among the 51 DEGs identified, the literature reports indicated several obesity-related genes, including NDUFS6, LAMA3, NCALD, QKI, UTRN, RNGTT, FGFRL1, PNPLA2, IGFBP5, BIN1, CDH13, PRKCD, PRKAR1B, PCYT2, SIRT7, CDS2, and NFIB. Changes in the expression of these genes may be associated with abdominal fat deposition in broilers. For example, NDUFS6 is involved in maintaining mitochondrial function and cell viability, indirectly regulating adipocyte development and functional maintenance [42]. LAMA3 is associated with adipocyte differentiation [33]. NCALD is a potential hippocampal memory correlate linked to obesity in rats [43], and Lu et al. [44] demonstrated that QKI, using mice as the experimental model, plays an important role in controlling the dynamic balance of adipose tissue metabolism. The UTRN gene is a candidate gene associated with intramuscular fat [45]. RNGTT is associated with fat deposition [46]. FGFRL1 expression may contribute to the development of obesity and its complications in humans [47]. Upregulation of PNPLA2 may represent a major compensatory event for visceral fat deposition associated with leptin [48]. Overexpression of IGFBP5 reduces the production of adipose-associated proteins and decreases intracellular lipid droplets [49]. BIN1 expression decreases in response to obesity and aging in mice [50]. CDH13 has been associated with visceral fat accumulation [51]. PRKCD may serve as a key target for obesity treatment [52]. Ji et al. [53] reported that PRKAR1B expression is regulated by endoplasmic reticulum stress and participates in lipolysis in grass carp; PCYT2 may be related to insulin resistance in humans, affecting obesity development [54]. SIRT7 regulates adipogenesis in adipocytes via PPAR-γ2 deacetylation [55]. Xu et al. [56] found that CDS2 isoforms carry 3′-UTR mutations and are highly expressed in chicken adipose tissue, and the NFIB complex with MLL1 can cooperate with KDM4D to promote the expression of key lipogenic regulators [57]. All 17 of these genes are associated with adipose tissue growth and development, supporting the reliability of the results and suggesting that SNPs located in these genes may influence abdominal fat deposition in broilers, warranting further investigation. Importantly, although other genes identified in this study have not been previously reported to be associated with adipose tissue growth and development, the SNPs within these genes should not be overlooked, as their potential roles in fat metabolism may not yet have been explored. However, species-specific differences also exist. The regulatory regions and SNP effects we observed in chickens may differ from those in mammals due to differences in genomic architecture and regulatory mechanisms. For example, while PPARG is a key adipogenic transcription factor across species, the specific SNPs affecting its expression or function can vary substantially between chickens and mammals [58,59]. This highlights the importance of species-specific studies for marker-assisted selection programs.

In addition, SNP loci may not only influence traits by regulating the expression of the genes in which they reside, but may also act at a distance to modulate the expression of target genes, thereby affecting phenotypic variation. In this study, the 2302 SNPs associated with broiler fat deposition were further analyzed in combination with 3D genomic (Hi-C) data from broiler adipose tissue, resulting in the identification of 15 SNPs that may remotely regulate the expression of target genes. These SNPs potentially affect 11 genes, including POSTN, GPC4, ST3GAL5, DOCK2, CORO1C, and APOBEC2. For example, loss of POSTN attenuates adipose tissue fibrosis and improves insulin resistance [60]. GPC4 expression is significantly higher in visceral fat than in subcutaneous fat, suggesting a role in adipose distribution [61]. ST3GAL5 is predominantly expressed in adipose stromal cells [62]. DOCK2 may serve as a therapeutic target for obesity-related diseases [63]. CORO1C is significantly enriched in various obesity-related pathways and GO functions [64], and APOBEC2 may promote lipid uptake and transport [65]. These findings collectively support the notion that SNPs can exert substantial effects on adipogenesis not only through local gene regulation but also via long-range regulation of distant target genes.

### 4.3. Pathways of SNPs Regulating Target Gene Expression

SNPs are abundant and scattered across different regions of the genome, which results in diverse pathways through which they can regulate gene expression [66,67,68]. In this study, SNPs were categorized according to their genomic locations into coding-region SNPs and non-coding-region SNPs to explore the mechanisms by which they influence gene expression.

For SNPs located in coding regions, they may affect gene expression by altering phosphorylation, glycosylation, protein hydrophobicity, protein secondary structure, codon translation rate, and mRNA secondary structure. Phosphorylation modifications participate in signaling and gene expression regulation during cell differentiation, influencing the cell cycle and regulating multiple metabolic pathways, including energy metabolism, glycolysis, and lipid metabolism [69]. Glycosylation modifications affect protein folding and stability, thereby influencing gene expression [70]. Protein hydrophobicity can impact protein structure, stability, and interactions with other molecules [71], while protein secondary structure, including α-helices, β-sheets, and β-turns, is crucial for protein stability, and alterations can significantly affect protein folding and function [72]. Codon translation rate can influence the speed of amino acid incorporation and protein folding, thereby modulating gene expression [73]. mRNA secondary structure affects gene expression by regulating mRNA stability, longevity, and the efficiency of transcription and translation [74].

Bioinformatics analysis of the eight coding-region SNPs revealed that they did not alter phosphorylation, glycosylation, protein hydrophobicity, or secondary structure. However, analysis of codon translation rates and mRNA secondary structure showed that the rs315605586 mutation accelerated codon translation, whereas rs15674853 and rs314812968 slowed it. Additionally, rs314812968 and rs316935384 increased mRNA secondary structure stability, while rs15679530 decreased it. Notably, rs314812968 both decreased codon translation rate and increased mRNA secondary structure stability; a highly stable mRNA structure can hinder ribosome binding, reducing translation efficiency. Although SYT15, where rs314812968 is located, has not been directly linked to adipogenesis, it has been associated with body weight, glucose tolerance, and lipid levels in rats [75]. Therefore, rs314812968 may influence abdominal fat deposition in broilers by slowing translation and reducing gene expression. SNPs in both coding and non-coding regions can also affect gene expression by altering splice site activity, including exonic, 5′, and 3′ splice sites. Changes in exonic splice sites can generate alternative protein isoforms, while alterations in 5′ or 3′ splice sites may affect mRNA stability, transport, or translation [76]. Analysis of the 201 SNPs located on differentially expressed genes revealed that none caused splice site changes, likely due to the stringent screening criteria applied.

Compared with coding-region SNPs, non-coding-region SNPs can regulate gene expression by altering transcription factor (TF) binding sites. Such alterations may change TF-DNA binding affinity [77] or modify selective TF binding across genes or promoter/enhancer regions [78], thereby influencing transcriptional activity. In this study, non-coding-region SNPs were classified into those regulating their own genes and those acting on distal genes.

Further analysis showed that among the 193 non-coding SNPs on differentially expressed genes, 192 altered TF binding sites, and all 193 led to differences in specific TF binding. Classical adipose-associated TFs, including PPARG [79], SREBP [80], CEBP [81], NFKB [82], RXR [83], AP2 [84], CREB [85], and FOXO [86], were used to validate these results. Of the 193 SNPs, 116 segregated or bound to adipose-related TFs before and after mutation, with 55 located in genes previously associated with adiposity. These findings indicate that most identified SNPs are likely relevant to abdominal fat deposition. It should be noted that only a subset of adipose-related TFs was examined, and untested TFs may also interact with these SNPs. Thus, SNPs not shown to bind adipose-related TFs may still affect abdominal fat by altering TF binding activity and modulating gene expression, leading to differential expression in adipose tissues of high- and low-fat broilers.

Moreover, the 15 SNPs identified through 3D genomic (Hi-C) analysis, which may remotely regulate target genes, also altered TF binding sites and affinities. This suggests that these SNPs may change genomic interaction regions, modifying regulatory relationships with distal enhancers or promoters and resulting in differential gene expression. This hypothesis is supported by prior studies; for instance, Santiago et al. [78] found that promoters can preferentially recruit STAT1/2 and IRF to regulate distal gene expression, Zhu et al. [87] reported that rs1440702 affects distal SOX6 expression by modifying TCF4 binding to enhancers, and Yu et al. [88] showed that rs7198799 regulates ZFP90 expression via NFATC2 binding.

In summary, SNPs that regulate target gene expression were analyzed in conjunction with phenotypic data, confirming that these SNPs have tangible effects on abdominal fat deposition in broilers and supporting the reliability of our findings. The SNP regulatory mechanisms identified in this study involve not only the effects of coding regions on translation efficiency and mRNA structural stability, but also the contributions of non-coding regions through transcription factor binding and long-range regulatory interactions. The complexity of these mechanisms suggests that abdominal fat deposition in broilers is governed by multi-layered genetic regulatory networks. Future functional validation experiments—such as CRISPR/Cas9-mediated gene editing or transcription factor binding assays—will be crucial to confirm the regulatory roles of these SNPs and facilitate their application in molecular breeding of broilers.

## 5. Conclusions

In this study, we screened and identified a set of key SNPs associated with abdominal fat deposition in broilers, integrating transcriptome, genome resequencing, and three-dimensional genomic data. A total of 213 SNP loci were found to be significantly related to abdominal fat deposition. These polymorphisms may influence the expression of target genes by altering codon translation efficiency, modifying the stability of mRNA secondary structures, and affecting the activity of transcription factor binding sites, thereby regulating abdominal fat accumulation in broilers.

## Figures and Tables

**Table 1:** Difference in SNP between high fat and low fat broilers (Part).

Chr	rs	WT	MT	FL_1	FL_2	FL_3	FL_4	FL_5	LL_1	LL_2	LL_3	LL_4	LL_5
1	337683	A	G	G/G	G/A	G/G	G/G	G/G	A/A	A/A	A/A	A/A	A/A
2	3908887	T	C	T/T	T/T	T/T	T/T	T/T	C/C	C/C	C/C	C/C	C/C
3	2127089	C	A	A/A	A/A	A/A	A/A	A/A	C/C	C/C	C/C	C/C	C/C
4	31488349	C	T	T/T	T/T	T/T	T/T	T/T	C/C	C/C	C/T	C/C	C/C
5	9960422	G	A	G/G	G/G	G/G	G/G	G/G	A/A	A/A	A/A	A/A	A/A
6	12977377	G	T	T/T	T/T	T/T	T/T	T/T	G/G	G/G	G/G	G/G	G/T
7	2716815	A	G	G/G	G/G	G/G	A/G	G/G	A/A	A/A	A/A	A/A	A/A
8	27654376	G	T	G/G	G/G	G/G	G/G	G/G	T/T	T/T	T/T	T/T	G/T
9	3548057	G	A	G/A	G/G	G/G	G/G	G/G	A/A	A/A	A/A	A/A	A/A
Z	70803755	A	C	A/A	A/A	A/A	A/A	A/A	C/C	C/C	C/C	C/C	C/C

**Table 2:** Distal target genes regulated by SNP.

Chr	rs	rsID	WT	MT	Gene
1	173675555	rs733601174	C	T	POSTN
4	3022801	rs316329973	G	A	GPC4, HS6ST2
4	85315858	rs739468529	T	C	ST3GAL5
4	85317113	rs313259902	A	T	ST3GAL5
4	85317787	rs316888112	G	A	ST3GAL5
4	85318002	rs14501054	C	T	ST3GAL5
4	85318751	rs741259739	C	T	ST3GAL5
4	85321374	rs735918912	G	A	ST3GAL5
4	89576514	rs15645705	G	A	C20orf194
5	51579773	rs317162307	A	G	ASPG
7	23241529	rs739919594	T	C	TNS1
10	17802386	rs741174402	A	T	CHSY1
13	5392030	rs14990733	T	C	DOCK2
15	8176792	rs315551857	A	G	CORO1C
26	3856885	rs315369512	G	C	APOBEC2

**Table 3:** Codon translation rate analysis.

rsID	WT	MT	CAI Value Before Mutation	CAI Value After Mutation	Gene
rs315605586	C	T	0.75	0.76	PAK3
rs15674853	T	C	0.78	0.77	STBD1
rs314812968	C	T	0.76	0.75	SYT15

The CAI value is the codon adaptation index, which refers to the adaptation coefficient when all codons encoding the protein use the optimal codon relative to the gene. The CAI value is between 0 and 1, and the higher the CAI value, the stronger the adaptability, thus improving the expression efficiency of the gene.

**Table 4:** Comparison of the difference in transcription factor binding sites before and after mutation.

rs	rsID	WT	MT	Specific Transcription Factor Binding Sites Before Mutation	Specific Transcription Factor Binding Sites After Mutation
1:173675555	rs733601174	C	T	PLAGL2, Plagl1, PLAG1, ZNF692, Zbtb41, Zfp711, ZNF134, ZNF707, Hnf4g, ZKSCAN3, ZBTB6, ZNF454	Prdm5, Zfp37, Hes7, ZNF135, Zfp691, TFAP2E
4:3022801	rs316329973	G	A	Zfp3, Zbtb20, Zfp184, Znf431	AR, Dmrtb1, ZNF189, Mafb
4:85315858	rs739468529	T	C		Zfp689, Rfx7, Hic1, Rfx4, ZNF527, ZNF582, RFX5, RFX2, Rfx3
4:85317113	rs313259902	A	T	FOXC1, Pax5	REST, Sall2
4:85317787	rs316888112	G	A	ZNF677	POU2F2, POU5F1, POU1F1, POU3F4, ZNF84, MAFF, POU6F1, PATZ1, Pou2f1, POU2F3, POU3F2, ZNF394, FEZF1, Foxm1, POU3F1, ZNF260
4:85318002	rs14501054	C	T	SREBF2, Rara, Nkx2-2, NKX2-1, RARG	YY1, MXI1, Prdm5, RFX5, BCL11A, Rfx2, Rfx1
4:85318751	rs741259739	C	T	PAX5, Nr1h4, ZNF791, ZNF383, FERD3L, ZNF681, ATF1	ZNF136, ZNF324, Npas4
4:85321374	rs735918912	G	A	JUND, NFE2, Npas4, JUNB	Sox4, ZNF768, ZNF212, Sox11, SOX3, ZNF652, SOX10, AR, ZNF223
4:89576514	rs15645705	G	A	HIC2, Mtf1, HIC1, Nr1h4, ATF6, ZNF141, ZNF93, ATF6B, Zbtb41	RFX3, ZNF429, THAP1, Rfx4, ZNF320, Npas4
5:51579773	rs317162307	A	G	ZNF35, ZNF280A, Zfp583	ZNF212, SOX18, ZNF180, Rela
7:23241529	rs739919594	T	C	ZNF3, ZNF768	MYBL1, ESR2, Myb, Mybl2, ZNF324
10:17802386	rs741174402	A	T	NR1D2	IRF4, Zfp41, Zfp287, Zfp768
13:5392030	rs14990733	T	C	ZIM3, Stat6, ZNF274	RELA, NFKB1, REL
15:8176792	rs315551857	A	G	PITX2, Myb	GSX2, GSX1, PBX4, Lhx2, POU6F2, Uncx, NKX6-2, LBX1, PRRX2, SHOX, ISX, HESX1, LHX9, ARX, VENTX, Pax6, PROP1, MEIS2, RORA, PRRX1, PAX4, MIXL1, NKX6-1, Nobox, HOXC4, NOTO, HOXD13, Nkx6-3, ZNF396, Lhx8, ESX1, LHX6, VSX1, Hlx, ZFHX2, En2, Phox2b, Phox2a, DLX3, Lhx1, RORC, Shox2, RAX2, Rhox6, HOXC13, MEOX1, EMX1, RAX, Otp, Gbx1
26:3856885	rs315369512	G	C	PAX6, ZNF621, ZNF768	THAP1, FOXO3, ZNF75A, Zfp770

**Table 5:** Comparison of the difference in affinity of transcription factors before and after mutation.

rs	rsID	WT	MT	Specific Transcription Factor Binding Sites Before Mutation	Specific Transcription Factor Binding Sites After Mutation
1:173675555	rs733601174	C	T	HNF4, COUP, USF_C	ZID
4:3022801	rs316329973	G	A	ZID	PAX2, CEBP_C, CEBPB, IRF1, CHOP, R
4:85315858	rs739468529	T	C	BRACH, GATA3, EVI1, SRY	STAF, PAX5, RFX1, CMYB
4:85317113	rs313259902	A	T	GATA1, CREL, CDP, S8, ISRE, IRF1, IRF2, HFH1, CEBP, PBX1	OCT1_06, HNF1_C, E4BP4, VBP, HLF, EVI1, NRSF, CMYB
4:85317787	rs316888112	G	A	GR, AHRARNT, IRF2, PAX5	EVI1
4:85318002	rs14501054	C	T	CMYB, MYB, VMYB	COMP1, RFX1, AP1FJ
4:85318751	rs741259739	C	T	ER, T3R, P300	USF, CDPCR3, MZF1, GATA1, GATA2, GATA3
4:85321374	rs735918912	G	A	PAX5, STAF, NRF2, ISRE	MZF1, HNF4, GR, CDXA
4:89576514	rs15645705	G	A	CMYB, E2, E2F, IK1	CDPCR3, CREB, USF_C, ER, GRE_C
5:51579773	rs317162307	A	G	NFKB_C, NFKAPPAB, GR, R, MYCMAX, LMO2COM, AML1	TAXCREB, VMYB
7:23241529	rs739919594	T	C	TAL1ALPHAE47, TAL1BETAITF2, GATA1, SRY	GR, GRE_C, COMP1
10:17802386	rs741174402	A	T	GRE_C, EVI1, CREL, USF, SP1	COMP1, IK3, NFE2, ELK1
13:5392030	rs14990733	T	C	POLY_C, EVI1	STAT1, CREL, DELTAEF1, STAT, CAP
15:8176792	rs315551857	A	G	PBX1, CREBP1	RORA1, ER
26:3856885	rs315369512	G	C	OLF1, CAAT	CEBPA, CEBP, GFI1, MZF1

## Data Availability

All data is included in this paper.

## Supplementary Materials

The following supporting information can be downloaded at: https://www.mdpi.com/article/10.3390/ani15192811/s1, Table S1: SNP located in differentially expressed genes; Table S2: Comparison of the difference in transcription factor binding sites before and after mutation; Table S3: Comparison of the difference in affinity of transcription factors before and after mutation.
