# Supplementary material for: Identification and Pathway Analysis of SNP Loci Affecting Abdominal Fat Deposition in Broilers
Source: Animals (Basel). 2025 Sep 26;15(19):2811. doi: 10.3390/ani15192811 (PMC12524246; doi:10.3390/ani15192811)
Supplement: Supplementary file 1 [file animals-15-02811-s001.zip › animals-3879907-supplementary.pdf]

`

**Table S1**. SNP located in differentially expressed genes

| Chr | rs | rsID | WT | MT | ∣Δ Allele frequency∣ | χ2 | Region annotation | Gene ID |
|---|---|---|---|---|---|---|---|---|
| 1 | 27173461 | rs15216781 | C | T | 0.875 | 274.822（P<0.001） | Intron | DOCK4 |
| 1 | 27184061 | rs315145701 | T | C | 0.887 | 272.79（P<0.001） | Intron | DOCK4 |
| 1 | 27184104 | rs314397809 | C | T | 0.875 | 267.247（P<0.001） | Intron | DOCK4 |
| 1 | 27184143 | rs13842215 | T | C | 0.873 | 269.396（P<0.001） | Intron | DOCK4 |
| 1 | 27184545 | rs735711891 | C | T | 0.885 | 276.963（P<0.001） | Intron | DOCK4 |
| 1 | 27196512 | rs13842248 | C | T | 0.919 | 296.491（P<0.001） | Intron | DOCK4 |
| 1 | 27196619 | rs317694843 | T | C | 0.785 | 248.381（P<0.001） | Intron | DOCK4 |
| 1 | 27237136 | rs315047313 | C | T | 0.731 | 236.975（P<0.001） | Intron | DOCK4 |
| 1 | 27240573 | rs317433662 | T | G | 0.700 | 215.726（P<0.001） | Intron | DOCK4 |
| 1 | 27243212 | rs317015848 | T | G | 0.706 | 215.84（P<0.001） | Intron | DOCK4 |
| 1 | 27283069 | rs314576853 | A | T | 0.700 | 214.07（P<0.001） | Intron | DOCK4 |
| 1 | 27406542 | rs312848604 | G | A | 0.784 | 274.814（P<0.001） | Intron | DOCK4 |
| 1 | 60619217 | rs317224719 | C | T | 0.751 | 221.616（P<0.001） | Intron | ERC1 |
| 1 | 83781180 | rs314346696 | T | C | 0.836 | 298.868（P<0.001） | Intron | QTRT2 |
| 1 | 176289110 | rs313999630 | A | C | 0.720 | 211.879（P<0.001） | Intron | B3GLCT |
| 1 | 176289152 | rs317629975 | A | C | 0.715 | 212.128（P<0.001） | Intron | B3GLCT |
| 1 | 176299324 | rs14922464 | A | T | 0.708 | 193.425（P<0.001） | Intron | B3GLCT |
| 1 | 176306216 | rs15512540 | C | T | 0.707 | 208.985（P<0.001） | Intron | B3GLCT |

（To be continued on next page）

（Continued from previous page）

| Chr | rs | rsID | WT | MT | ∣Δ Allele frequency∣ | χ2 | Region annotation | Gene ID |
|---|---|---|---|---|---|---|---|---|
| 1 | 176307593 | rs315526298 | G | A | 0.713 | 212.709（*P*<0.001） | Intron | *B3GLCT* |
| 1 | 176315868 | rs313356458 | C | T | 0.741 | 241.031（*P*<0.001） | Upstream region | *B3GLCT* |
| 1 | 187090099 | rs738182074 | C | T | 0.747 | 261.577（*P*<0.001） | Intron | *VSTM5* |
| 1 | 187091008 | rs316526526 | C | G | 0.719 | 221.506（*P*<0.001） | 3′UTR | *MED17* |
| 1 | 196509446 | rs316211752 | C | T | 0.806 | 257.56（*P*<0.001） | Intron | *ARHGEF17* |
| 1 | 196522180 | rs313962930 | G | A | 0.736 | 223.082（*P*<0.001） | Intron | *ARHGEF17* |
| 1 | 196522212 | rs734842753 | G | A | 0.776 | 244.711（*P*<0.001） | Intron | *ARHGEF17* |
| 1 | 196530963 | rs16004403 | A | G | 0.785 | 248.037（*P*<0.001） | Intron | *ARHGEF17* |
| 1 | 196549561 | rs317881329 | C | G | 0.720 | 202.707（*P*<0.001） | Intron | *ARHGEF17* |
| 1 | 196557882 | rs14939151 | A | G | 0.762 | 228.865（*P*<0.001） | Intron | *ARHGEF17* |
| 1 | 196557890 | rs316655562 | G | C | 0.765 | 230.758（*P*<0.001） | Intron | *ARHGEF17* |
| 1 | 196573425 | rs316889139 | T | G | 0.751 | 223.199（*P*<0.001） | Intron | *ARHGEF17* |
| 1 | 196573490 | rs314830954 | C | T | 0.736 | 212.625（*P*<0.001） | Intron | *ARHGEF17* |
| 1 | 196574056 | rs14939165 | A | T | 0.719 | 200.512（*P*<0.001） | Intron | *ARHGEF17* |
| 1 | 196580813 | rs314968551 | C | T | 0.718 | 213.546（*P*<0.001） | Intron | *ARHGEF17* |
| 1 | 196580993 | rs1058415596 | C | T | 0.754 | 238.042（*P*<0.001） | Intron | *ARHGEF17* |
| 1 | 196581449 | rs313415787 | G | A | 0.755 | 233.317（*P*<0.001） | Intron | *ARHGEF17* |
| 1 | 196582730 | rs1059181375 | A | G | 0.770 | 247.638（*P*<0.001） | Intron | *ARHGEF17* |
| 1 | 196585427 | rs313837380 | G | A | 0.755 | 237.831（*P*<0.001） | Intron | *ARHGEF17* |
| 1 | 196603689 | rs14939226 | G | A | 0.825 | 281.65（*P*<0.001） | Intron | *ARHGEF17* |
| 1 | 196603917 | rs14939230 | T | G | 0.794 | 255.167（*P*<0.001） | Intron | *ARHGEF17* |
| 1 | 196603944 | rs14939231 | C | T | 0.788 | 255.167（*P*<0.001） | Intron | *ARHGEF17* |
| 1 | 196604150 | rs315994985 | C | A | 0.791 | 255.167（*P*<0.001） | Intron | *ARHGEF17* |
| 1 | 196607410 | rs13998573 | C | T | 0.809 | 261.577（*P*<0.001） | Intron | *ARHGEF17* |
| 1 | 196607763 | rs317696844 | C | G | 0.794 | 261.577（*P*<0.001） | Intron | *ARHGEF17* |

（To be continued on next page）

（Continued from previous page）

| Chr | rs | rsID | WT | MT | ∣Δ Allele frequency∣ | χ2 | Region annotation | Gene ID |
|---|---|---|---|---|---|---|---|---|
| 2 | 85982181 | rs14945407 | T | A | 0.711 | 259.357（P<0.001） | Upstream region | NDUFS6 |
| 2 | 103139175 | rs313638395 | G | A | 0.733 | 227.148（P<0.001） | Expressed region | LAMA3 |
| 2 | 103139584 | rs314080876 | T | G | 0.729 | 236.926（P<0.001） | Intron | LAMA3 |
| 2 | 103140193 | rs80730241 | C | T | 0.751 | 247.38（P<0.001） | Intron | LAMA3 |
| 2 | 103141420 | rs80654245 | C | T | 0.622 | 192.245（P<0.001） | Intron | LAMA3 |
| 2 | 128794002 | rs734475775 | C | G | 0.719 | 278.213（P<0.001） | Intron | NCALD |
| 2 | 128811387 | rs315917362 | G | A | 0.731 | 281.65（P<0.001） | Intron | NCALD |
| 3 | 3070317 | rs315618371 | T | C | 0.710 | 213.017（P<0.001） | Intron | ASB3，MALL |
| 3 | 31980122 | rs3386518642 | A | G | 0.739 | 252.521（P<0.001） | Intron | RMDN2 |
| 3 | 31980198 | rs318017131 | C | G | 0.736 | 255.884（P<0.001） | Intron | RMDN2 |
| 3 | 44253181 | rs314758575 | G | A | 0.842 | 251.445（P<0.001） | Intron | QKI |
| 3 | 44286804 | rs314559836 | C | T | 0.750 | 232.046（P<0.001） | Intron | QKI |
| 3 | 44286842 | rs317987997 | G | C | 0.732 | 218.809（P<0.001） | Intron | QKI |
| 3 | 44309828 | rs316230214 | G | C | 0.814 | 280.636（P<0.001） | Intron | QKI |
| 3 | 44312747 | rs317042042 | C | T | 0.700 | 198.737（P<0.001） | Intron | QKI |
| 3 | 46102485 | rs315830313 | G | A | 0.748 | 255.894（P<0.001） | Intron | UTRN |
| 3 | 46104342 | rs317957463 | C | T | 0.767 | 280.36（P<0.001） | Intron | UTRN |
| 3 | 75849394 | rs314111651 | C | T | 0.714 | 239.984（P<0.001） | Intron | RNGTT |
| 4 | 13403229 | rs313026403 | C | T | 0.822 | 257.782（P<0.001） | Intron | PAK3 |
| 4 | 13410951 | rs312919885 | T | C | 0.832 | 274.684（P<0.001） | Intron | PAK3 |
| 4 | 13411609 | rs315605586 | C | T | 0.826 | 261.247（P<0.001） | Expressed region | PAK3 |
| 4 | 13412185 | rs14041540 | T | C | 0.830 | 268.652（P<0.001） | Intron | PAK3 |
| 4 | 13412706 | rs316916496 | G | T | 0.813 | 264.295（P<0.001） | Intron | PAK3 |
| 4 | 49898600 | rs314633821 | G | A | 0.728 | 228.136（P<0.001） | 3′UTR | STBD1 |
| 4 | 49898909 | rs15674853 | T | C | 0.749 | 247.508（P<0.001） | Expressed region | STBD1 |

（To be continued on next page）

（Continued from previous page）

| Chr | rs | rsID | WT | MT | ∣Δ Allele frequency∣ | χ2 | Region annotation | Gene ID |
|---|---|---|---|---|---|---|---|---|
| 4 | 85072848 | rs316234116 | G | A | 0.707 | 193.681（*P*<0.001） | Intron | *FGFRL1* |
| 4 | 85080308 | rs312880760 | A | G | 0.758 | 269.784（*P*<0.001） | Intron | *FGFRL1* |
| 4 | 85126506 | rs313768959 | T | G | 0.712 | 200.937（*P*<0.001） | Intron | *FGFRL1* |
| 4 | 85163257 | rs316272980 | T | C | 0.776 | 229.423（*P*<0.001） | Intron | *FGFRL1* |
| 4 | 85174363 | rs315105277 | A | G | 0.716 | 248.889（*P*<0.001） | Intron | *FGFRL1* |
| 4 | 85174958 | rs739153977 | G | A | 0.870 | 271.771（*P*<0.001） | Intron | *FGFRL1* |
| 4 | 85174966 | rs14416983 | A | G | 0.870 | 271.771（*P*<0.001） | Intron | *FGFRL1* |
| 4 | 85186151 | rs317436117 | A | G | 0.746 | 208.136（*P*<0.001） | Intron | *FGFRL1* |
| 4 | 85186566 | rs315254872 | G | A | 0.837 | 248.106（*P*<0.001） | Intron | *FGFRL1* |
| 4 | 85186726 | rs312661958 | G | T | 0.897 | 287.699（*P*<0.001） | Intron | *FGFRL1* |
| 4 | 85186875 | rs317783073 | C | T | 0.884 | 278.507（*P*<0.001） | Intron | *FGFRL1* |
| 4 | 85186906 | rs316596701 | G | A | 0.817 | 264.742（*P*<0.001） | Intron | *FGFRL1* |
| 4 | 85188198 | rs315646870 | G | A | 0.782 | 229.508（*P*<0.001） | Intron | *FGFRL1* |
| 4 | 85206627 | rs314840589 | A | C | 0.741 | 209.071（*P*<0.001） | Intron | *FGFRL1* |
| 4 | 85215012 | rs314029489 | A | G | 0.720 | 203.922（*P*<0.001） | Intron | *FGFRL1* |
| 4 | 85218175 | rs317961268 | A | G | 0.836 | 268.178（*P*<0.001） | Intron | *FGFRL1* |
| 4 | 85382858 | rs733195637 | G | T | 0.835 | 242.132（*P*<0.001） | Downstream region | *ST3GAL5* |
| 4 | 89114648 | rs316478876 | A | C | 0.739 | 216.489（*P*<0.001） | Intron | *DNAAF9* |
| 4 | 89116915 | rs317056444 | T | C | 0.638 | 196.262（*P*<0.001） | Intron | *DNAAF9* |
| 4 | 89121169 | rs312458031 | A | G | 0.540 | 171.719（*P*<0.001） | Intron | *DNAAF9* |
| 4 | 89125298 | rs14417266 | C | G | 0.717 | 202.174（*P*<0.001） | Intron | *DNAAF9* |
| 5 | 15985047 | rs14417271 | C | A | 0.706 | 214.046（*P*<0.001） | Intron | *PNPLA2* |
| 5 | 22365615 | rs431846683 | T | G | 0.969 | 318.202（*P*<0.001） | Intron | *TSPAN18* |
| 5 | 28043501 | rs316576299 | A | G | 0.772 | 264.834（*P*<0.001） | Intron | *SMOC1* |
| 5 | 56580820 | rs16087237 | C | T | 0.763 | 249.279（*P*<0.001） | Intron | *SAMD4A* |

（To be continued on next page）

（Continued from previous page）

| Chr | rs | rsID | WT | MT | \|Δ Allele frequency\| | χ2 | Region annotation | Gene ID |
|---|---|---|---|---|---|---|---|---|
| 5 | 56593944 | rs16087254 | A | G | 0.725 | 225.21（*P*<0.001） | Intron | *SAMD4A* |
| 5 | 56596059 | rs14228382 | T | G | 0.767 | 248.228（*P*<0.001） | Intron | *SAMD4A* |
| 5 | 56596084 | rs314357717 | T | C | 0.779 | 258.91（*P*<0.001） | Intron | *SAMD4A* |
| 5 | 56615492 | rs313700011 | T | G | 0.807 | 243.812（*P*<0.001） | Intron | *SAMD4A* |
| 5 | 56616348 | rs738189398 | T | C | 0.785 | 257.416（*P*<0.001） | Intron | *SAMD4A* |
| 5 | 56618857 | rs314686498 | G | A | 0.821 | 258.226（*P*<0.001） | Intron | *SAMD4A* |
| 6 | 3440054 | rs316805216 | A | G | 0.734 | 248.889（*P*<0.001） | Expressed region | *SYT15* |
| 6 | 3443192 | rs314812968 | C | T | 0.713 | 252.012（*P*<0.001） | Expressed region | *SYT15* |
| 6 | 5075200 | rs16248915 | T | A | 0.847 | 295.779（*P*<0.001） | Intron | *TSPAN14* |
| 6 | 5075240 | rs16248917 | T | C | 0.844 | 288.636（*P*<0.001） | Intron | *TSPAN14* |
| 6 | 5076732 | rs16263103 | C | A | 0.841 | 288.636（*P*<0.001） | Intron | *TSPAN14* |
| 6 | 5078632 | rs313237810 | G | A | 0.721 | 211.281（*P*<0.001） | Intron | *TSPAN14* |
| 6 | 5081150 | rs734297511 | A | C | 0.737 | 220.337（*P*<0.001） | Intron | *TSPAN14* |
| 6 | 5084601 | rs733775780 | G | A | 0.721 | 214.484（*P*<0.001） | Intron | *TSPAN14* |
| 6 | 5100033 | rs317193066 | A | G | 0.734 | 211.056（*P*<0.001） | 5′UTR | *TSPAN14* |
| 6 | 10310975 | rs735459861 | A | G | 0.740 | 235.365（*P*<0.001） | Intron | *PAPSS2* |
| 7 | 23237809 | rs739305450 | G | A | 0.738 | 245.799（*P*<0.001） | Intron | *IGFBP5* |
| 7 | 23238115 | rs16302600 | A | G | 0.754 | 235.682（*P*<0.001） | Intron | *IGFBP5* |
| 7 | 23238248 | rs13644638 | G | A | 0.800 | 251.812（*P*<0.001） | Intron | *IGFBP5* |
| 7 | 23238382 | rs15500309 | A | G | 0.759 | 248.889（*P*<0.001） | Intron | *IGFBP5* |
| 7 | 23239760 | rs10722908 | C | T | 0.744 | 248.889（*P*<0.001） | Intron | *IGFBP5* |
| 7 | 23241529 | rs317910469 | T | C | 0.761 | 235.753（*P*<0.001） | Intron | *IGFBP5* |
| 7 | 23685239 | rs15500320 | A | G | 0.772 | 274.814（*P*<0.001） | Intron | *BIN1* |
| 7 | 23691084 | rs315259024 | T | C | 0.910 | 296.772（*P*<0.001） | Intron | *BIN1* |
| 7 | 23691185 | rs318238166 | C | T | 0.927 | 299.362（*P*<0.001） | Intron | *BIN1* |

（To be continued on next page）

（Continued from previous page）

| Chr | rs | rsID | WT | MT | ∣Δ Allele frequency∣ | χ2 | Region annotation | Gene ID |
|---|---|---|---|---|---|---|---|---|
| 7 | 23691531 | rs314239110 | C | T | 0.820 | 260.776（$P$<0.001） | Intron | *BIN1* |
| 7 | 23705417 | rs15612774 | A | G | 0.788 | 248.889（$P$<0.001） | Intron | *BIN1* |
| 7 | 23706503 | rs315549948 | G | A | 0.927 | 297.602（$P$<0.001） | Intron | *BIN1* |
| 7 | 23707501 | rs735858829 | A | G | 0.885 | 302.614（$P$<0.001） | Intron | *BIN1* |
| 7 | 23713609 | rs313639521 | C | T | 0.819 | 295.779（$P$<0.001） | Intron | *BIN1* |
| 7 | 23715340 | rs14693158 | T | C | 0.742 | 256.168（$P$<0.001） | Intron | *BIN1* |
| 7 | 23719007 | rs80768722 | A | G | 0.848 | 272.828（$P$<0.001） | Intron | *BIN1* |
| 7 | 23722085 | rs16691660 | T | C | 0.763 | 230.813（$P$<0.001） | Intron | *BIN1* |
| 7 | 23729993 | rs16691659 | G | A | 0.775 | 278.213（$P$<0.001） | Intron | *BIN1* |
| 7 | 23732444 | rs14507727 | A | G | 0.738 | 242.74（$P$<0.001） | Intron | *BIN1* |
| 7 | 23732471 | rs315784018 | T | C | 0.844 | 272.812（$P$<0.001） | Intron | *BIN1* |
| 7 | 23732517 | rs316191687 | A | G | 0.782 | 265.83（$P$<0.001） | Intron | *BIN1* |
| 7 | 23741246 | rs80587559 | C | T | 0.713 | 255.167（$P$<0.001） | Intron | *BIN1* |
| 7 | 23743401 | rs739426901 | G | A | 0.703 | 252.012（$P$<0.001） | Intron | *BIN1* |
| 7 | 23749916 | rs80730603 | A | C | 0.804 | 245.075（$P$<0.001） | Intron | *BIN1* |
| 7 | 23752118 | rs318220524 | T | A | 0.774 | 228.073（$P$<0.001） | Intron | *BIN1* |
| 7 | 23753048 | rs739403822 | G | A | 0.728 | 271.452（$P$<0.001） | Intron | *BIN1* |
| 7 | 23754967 | rs80692083 | T | C | 0.837 | 243.81（$P$<0.001） | Intron | *BIN1* |
| 7 | 23755992 | rs315864220 | C | T | 0.844 | 272.936（$P$<0.001） | Intron | *BIN1* |
| 7 | 23756991 | rs314772558 | G | A | 0.759 | 258.355（$P$<0.001） | Intron | *BIN1* |
| 7 | 23756996 | rs313942842 | A | G | 0.759 | 258.355（$P$<0.001） | Intron | *BIN1* |
| 8 | 2866334 | rs317104670 | C | T | 0.736 | 209.842（$P$<0.001） | Intron | *DENND1B* |
| 10 | 7222733 | rs733428170 | T | A | 0.752 | 246.636（$P$<0.001） | Intron | *MYO1E* |
| 10 | 7224607 | rs80735721 | G | A | 0.702 | 204.367（$P$<0.001） | Intron | *MYO1E* |
| 10 | 7233627 | rs15679530 | C | T | 0.767 | 255.954（$P$<0.001） | Expressed region | *MYO1E* |

（To be continued on next page）

（Continued from previous page）

| Chr | rs | rsID | WT | MT | ∣Δ Allele frequency∣ | χ2 | Region annotation | Gene ID |
|---|---|---|---|---|---|---|---|---|
| 11 | 15476382 | rs15690110 | A | G | 0.751 | 277.315（*P*<0.001） | Intron | *GAN* |
| 11 | 15477314 | rs16519176 | C | T | 0.772 | 266.066（*P*<0.001） | Intron | *GAN* |
| 11 | 16237253 | rs731954564 | A | G | 0.891 | 278.213（*P*<0.001） | Intron | *CDH13* |
| 11 | 16311558 | rs316739697 | G | A | 0.941 | 306.797（*P*<0.001） | Intron | *CDH13* |
| 11 | 16311635 | rs318127048 | G | C | 0.950 | 318.197（*P*<0.001） | Intron | *CDH13* |
| 11 | 16323284 | rs314518447 | A | G | 0.928 | 295.987（*P*<0.001） | Intron | *CDH13* |
| 11 | 16348866 | rs14554663 | T | C | 0.934 | 306.797（*P*<0.001） | Intron | *CDH13* |
| 12 | 1727650 | rs15743050 | C | G | 0.733 | 222.132（*P*<0.001） | Intron | *PRKCD* |
| 12 | 11801847 | rs16527532 | A | G | 0.903 | 299.745（*P*<0.001） | Intron | *SETD5* |
| 12 | 11801904 | rs315866764 | T | C | 0.890 | 291.676（*P*<0.001） | Intron | *SETD5* |
| 12 | 11801963 | rs312861187 | G | A | 0.869 | 276.925（*P*<0.001） | Intron | *SETD5* |
| 12 | 11801979 | rs312491466 | A | G | 0.866 | 273.404（*P*<0.001） | Intron | *SETD5* |
| 12 | 11802112 | rs312592149 | T | A | 0.864 | 277.945（*P*<0.001） | Intron | *SETD5* |
| 12 | 11802772 | rs312973670 | G | A | 0.901 | 303.687（*P*<0.001） | Intron | *SETD5* |
| 12 | 11802862 | rs315588466 | T | C | 0.898 | 307.454（*P*<0.001） | Intron | *SETD5* |
| 12 | 11802865 | rs16530658 | T | C | 0.898 | 307.454（*P*<0.001） | Intron | *SETD5* |
| 12 | 11804201 | rs314184780 | A | G | 0.881 | 292.037（*P*<0.001） | Intron | *SETD5* |
| 12 | 11804663 | rs15776234 | A | G | 0.911 | 311.537（*P*<0.001） | Intron | *SETD5* |
| 12 | 11804863 | rs739635299 | C | A | 0.922 | 318.232（*P*<0.001） | Intron | *SETD5* |
| 12 | 19780775 | rs316811946 | C | T | 0.817 | 287.627（*P*<0.001） | Intron | *SRGAP3* |
| 12 | 19783140 | rs316188605 | A | C | 0.801 | 273.474（*P*<0.001） | Intron | *SRGAP3* |
| 12 | 19784461 | rs314985840 | A | G | 0.844 | 299.119（*P*<0.001） | Intron | *SRGAP3* |
| 12 | 19786586 | rs793931460 | C | T | 0.815 | 276.363（*P*<0.001） | Intron | *SRGAP3* |
| 12 | 19801364 | rs739919594 | G | T | 0.786 | 273.096（*P*<0.001） | Intron | *SRGAP3* |
| 13 | 10806986 | rs734759241 | T | C | 0.738 | 242.74（*P*<0.001） | Expressed region | *NHP2* |

（To be continued on next page）

（Continued from previous page）

| Chr | rs | rsID | WT | MT | ∣Δ Allele frequency∣ | χ2 | Region annotation | Gene ID |
|---|---|---|---|---|---|---|---|---|
| 14 | 1238758 | rs316991790 | A | G | 0.716 | 202.993（*P*<0.001） | Intron | *BFAR* |
| 14 | 2508316 | rs313792827 | C | A | 0.713 | 202.117（*P*<0.001） | Intron | *PRKAR1B* |
| 14 | 14628939 | rs312457584 | C | A | 0.708 | 262.498（*P*<0.001） | Intron | *VRK3* |
| 18 | 7916856 | rs313224745 | A | G | 0.731 | 258.355（*P*<0.001） | Intron | *ABCA8* |
| 18 | 9751313 | rs316935384 | A | G | 0.741 | 227.906（*P*<0.001） | Expressed region | *ENGASE* |
| 18 | 9947166 | rs740514701 | T | C | 0.769 | 218.994（*P*<0.001） | 3'UTR | *PCYT2* |
| 18 | 9950906 | rs316894769 | C | A | 0.782 | 238.462（*P*<0.001） | Intron | *PCYT2* |
| 18 | 9959147 | rs15864493 | C | G | 0.750 | 219.012（*P*<0.001） | Intron | *SIRT7* |
| 18 | 9960650 | rs314090469 | A | G | 0.815 | 254.964（*P*<0.001） | Intron | *SIRT7* |
| 19 | 6505623 | rs14617318 | A | G | 0.790 | 287.706（*P*<0.001） | Intron | *SPECC1* |
| 22 | 461024 | rs14617319 | C | T | 0.779 | 234.436（*P*<0.001） | Intron | *CDS2* |
| 27 | 3796945 | rs313296176 | G | A | 0.701 | 221.687（*P*<0.001） | Intron | *MAP3K14, FMNL1* |
| Z | 30960219 | rs313456709 | T | C | 0.511 | 114.902（*P*<0.001） | Intron | *MPDZ* |
| Z | 30982495 | rs730934738 | C | T | 0.728 | 252.012（*P*<0.001） | Intron | *MPDZ* |
| Z | 31467999 | rs312449117 | C | T | 0.759 | 262.096（*P*<0.001） | Intron | *NFIB* |
| Z | 31468208 | rs738431855 | A | G | 0.798 | 291.566（*P*<0.001） | Intron | *NFIB* |
| Z | 31468803 | rs3388220694 | T | C | 0.709 | 225.028（*P*<0.001） | Intron | *NFIB* |
| Z | 31469397 | rs317018855 | C | T | 0.716 | 230.813（*P*<0.001） | Intron | *NFIB* |
| Z | 31470828 | rs314490595 | T | C | 0.703 | 230.813（*P*<0.001） | Intron | *NFIB* |
| Z | 31471141 | rs313488012 | C | T | 0.774 | 269.503（*P*<0.001） | Intron | *NFIB* |
| Z | 31471295 | rs313387429 | T | C | 0.768 | 276.737（*P*<0.001） | Intron | *NFIB* |
| Z | 31474541 | rs735129014 | T | G | 0.753 | 264.834（*P*<0.001） | Intron | *NFIB* |
| Z | 31474926 | rs733539751 | T | C | 0.713 | 239.713（*P*<0.001） | Intron | *NFIB* |
| Z | 31475239 | rs16776397 | T | C | 0.747 | 255.675（*P*<0.001） | Intron | *NFIB* |
| Z | 31475464 | rs741291342 | A | T | 0.719 | 230.196（*P*<0.001） | Intron | *NFIB* |

（To be continued on next page）

`

(Continued from previous page)

| Chr | rs | rsID | WT | MT | ∣Δ Allele frequency∣ | χ2 | Region annotation | Gene ID |
|---|---|---|---|---|---|---|---|---|
| Z | 31476489 | rs316071695 | G | T | 0.713 | 233.75（P<0.001） | Intron | NFIB |
| Z | 31478846 | rs733148472 | A | C | 0.753 | 248.889（P<0.001） | Intron | NFIB |
| Z | 31481217 | rs736318169 | T | A | 0.728 | 236.716（P<0.001） | Intron | NFIB |
| Z | 31492476 | rs314955625 | A | T | 0.775 | 265.969（P<0.001） | Intron | NFIB |
| Z | 31492800 | rs313371449 | A | G | 0.709 | 225.028（P<0.001） | Intron | NFIB |
| Z | 31494181 | rs14778403 | A | G | 0.719 | 242.74（P<0.001） | Intron | NFIB |
| Z | 31494359 | rs312440942 | T | C | 0.703 | 233.75（P<0.001） | Intron | NFIB |
| Z | 31501598 | rs740312969 | T | C | 0.728 | 236.716（P<0.001） | Intron | NFIB |

`

**Table S2.** Comparison of the difference of transcription factor binding sites before and after mutation

| rs | rsID | WT | MT | Specific transcription factor binding site before mutation | Specific transcription factor binding site after mutation |
|---|---|---|---|---|---|
| 1:27173461 | rs15216781 | C | T | ZNF823, T, ZNF223, ZNF33B, TBX19 | BARX1, HOXB4, HOXD4, HOXD3, PDX1, BARX2, ISL1, HOXC4, HOXB5, NANOG, VAX2, EVX2, HOXB3, NKX1-1, HOXA3, HOXA5, HOXB6 |
| 1:27184061 | rs315145701 | T | C | LBX2, HLX, ZNF549, SMAD3, SMAD4, TLX2, HOXD11, WT1, SOX8, LHX3, FOXO1, SOX21, AHCTF1, HOXB7, NR2E3 | HOXB2, POU4F2, POU6F1, GLI3, NKX3-1 |
| 1:27184104 | rs314397809 | C | T | | SOX2, ZNF454, GLI2, ZFP319, NANOG, ZNF45, POU5F1, FOXP3 |
| 1:27184143 | rs13842215 | T | C | GTF3A, PPARG, ZNF708 | GCM1, EGR2, ZSCAN22, ZNF513, ZFP281, ZNF616, ZNF580, GCM2, ZNF281, ZNF468, VEZF1, KLF3, EGR1, ZFP770, PATZ1 |
| 1:27184545 | rs735711891 | C | T | BACH2, MAFK, MAFG, ZNF260, NFE2, T, NFE2L2, BCL11B, MAFB, MAF, ZSCAN4, ZNF554 | THRB, ZNF436 |
| 1:27196512 | rs13842248 | C | T | ZFP1, WT1, ZNF805, ZNF300, ZNF444, ZFP770, ZFP281, INSM1, ZSCAN10, CTCF, ZNF740, ZIC1, BHLHA15, ZFP740 | ZSCAN22, ZNF483, ZNF707 |
| 1:27196619 | rs317694843 | T | C | KLF4, TCF21, MYOD1, HAND2, ZNF780A, ZNF71 | ZNF519, ZNF619, ZNF566, PRDM9, ZFP536, ZNF12, SNAI1, ZNF263, TEAD1 |
| 1:27237136 | rs315047313 | C | T | ZNF808 | HOXA9, PBX2 |
| 1:27240573 | rs317433662 | T | G | SOX1, HOXA5, POU3F4, SOX10 | ZNF805, ZBTB44, ZNF558, NHLH2 |
| 1:27243212 | rs317015848 | T | G | ZNF586 | ZSCAN30, SMAD2, ZNF529, HES7, ZNF322, NFIC, ZNF490, ZNF436, PRDM9 |
| 1:27283069 | rs314576853 | A | T | IRF2, PRDM1, IRF1, IRF4 | |
| 1:27406542 | rs312848604 | G | A | | KLF1, ZNF274, BATF3, RELA |

（To be continued on next page）

`

（Continued from previous page）

| rs | rsID | WT | MT | Specific transcription factor binding site before mutation | Specific transcription factor binding site after mutation |
|---|---|---|---|---|---|
| 1:60619217 | rs317224719 | C | T | NKX3-1, NKX2-5, NKX2-3, NKX2-8, RARG, ZSCAN31, NKX2-2, CTCF, RARB, ZNF713 | FOXP3, ZFP1, SNAI1, TGIF1 |
| 1:83781180 | rs314346696 | T | C | FOXC2, ZNF585A, FOXI1, FOXJ2, ZFP74 | |
| 1:176289110 | rs313999630 | A | C | RUNX1, RUNX3, RARA, GFI1B, RUNX2, NKX2-5, ZNF547, NR2C1 | ZFP526, ZFP641, ZNF77, HNF4G, HNF4A |
| 1:176289152 | rs317629975 | A | C | OLIG3, ZNF211, TAL1, ZFP583, FIGLA, MAFK, ZNF273 | ZNF76, NFATC1, RELA, ZFP410, MEIS2, PKNOX2 |
| 1:176299324 | rs14922464 | A | T | HOXA5 | IRF9, ZNF287, ZNF713, ZNF585A, ZNF418 |
| 1:176306216 | rs15512540 | C | T | ZBTB48, INSM1, TBX20, FOXN2 | ZNF343, ELF5, SPIB |
| 1:176307593 | rs315526298 | G | A | IKZF1, AIRE, STAT2, ZNF45, ZNF24 | IRF1, ZIM3, ZFP28, AHCTF1, IRF8, ZNF527, NKX6-1, IRF9, IRF4 |
| 1:176315868 | rs313356458 | C | T | ZNF442 | SOX3, ESRRA, ESRRB, ATF3, NR5A2 |
| 1:187090099 | rs738182074 | C | T | CTCF | ELF3, IRF3, MZF1, ZNF276, IRX5, IRF4, IRX2 |
| 1:187091008 | rs316526526 | C | G | HSF4, HIF1A, DUXA, TBX1 | NKX2-8, ZNF615, CTCF, NR5A2, ESRRB, NR5A1, ESRRA, HINFP |
| 1:196509446 | rs316211752 | C | T | ZFP263, ZNF263, ZFP14 | ZFP672 |
| 1:196522180 | rs313962930 | G | A | NFATC4, NFATC2, NFATC3, IRF3, NFATC1, ZNF502, SOX17, STAT1, ZNF71, STAT2, ZNF140, ZNF33B, ZNF85, IRF4 | PRDM6, ZFP28, ZNF620 |
| 1:196522212 | rs734842753 | G | A | SOX10, ZFP697, RHOX11, PEG3, NFIB, ZNF805, NKX3-2, ZNF12, ZNF324, THRA, ZFP69B | OLIG3, RORA, TFAP4, SREBF1, PTF1A |
| 1:196530963 | rs16004403 | A | G | TCF21, ATOH1, TWIST2, NEUROG2, TFAP4, OLIG3, NEUROD2, BHLHE23, NEUROG1, OLIG1, BHLHE22, MAFF, ATOH7, ZFP41 | ZBTB20, MAF, ZFP14 |
| 1:196549561 | rs317881329 | C | G | ZNF33A, ZNF135, CTCF | NKX2-5, SP5, ZNF205, ZNF432, EGR2, ZNF267, PATZ1 |
| 1:196557882 | rs14939151 | A | G | FOXO3, SMAD7, FOXO1, RELA, ZNF334, FOXO6, ZFP672 | SALL3, STAT4, STAT5B, STAT1, BCL6B, ZNF534 |
| 1:196557890 | rs316655562 | G | C | SIX4, ZNF582 | ZNF793, TEAD2, TEAD4, ZNF419 |
| 1:196573425 | rs316889139 | T | G | FEZF1, ZNF45, ELF1, FOXK1, FOXG1 | ARID5B, KLF6, GLI3, ZNF264, KLF2, KLF1, ZNF90 |

（To be continued on next page）

（Continued from previous page）

| rs | rsID | WT | MT | Specific transcription factor binding site before mutation | Specific transcription factor binding site after mutation |
|---|---|---|---|---|---|
| 1:196573490 | rs314830954 | C | T | ZNF317, ZNF490, ZNF653 | ZFP184 |
| 1:196574056 | rs14939165 | A | T | ZNF75A, NFATC2, ZNF189, ZFP422 | ZNF528, RXRA, NR1H4, NR2E1, ZFP516, NR2C1, ZNF582, NR2C2, RARB, RARA, NR2F6, NR2F1, FEZF1, ESRRA, NR1I2, ESR1 |
| 1:196580813 | rs314968551 | C | T | OSR2, SP4, SMAD3, PATZ1, E2F7, SP2, ZSCAN30, ZNF37A, ZNF101, ZBTB6, CTCFL, ZNF571, ZNF460, SP3, ZNF180, ZNF281, ZNF267, ASCL1, WT1, ZNF529, ZNF707, ZNF467, ZNF415, KLF6, ZNF519, KLF15, RELA, ZNF132, ZNF93, SP5, TCF4, TFCP2L1 | HES7, NFIC, ZNF419, NFIA, ZIK1, ZFP513, ZNF555 |
| 1:196580993 | rs1058415596 | C | T | SMAD4 | |
| 1:196581449 | rs313415787 | G | A | CREB3L1, CLOCK, PRDM5, RORA, ZNF528, ZBTB24 | TFAP2A, ZNF345 |
| 1:196582730 | rs1059181375 | A | G | EVX2, HOXA11, CDX1, HOXC10, HOXA10, HOXA9, CDX4, HOXC12, HOXD10, HOXB9, HOXD11, HOXB13, HOXC11, HOXD9, HOXD13, HOXC9, HOXA13, HOXC13, HOXD12, HOXC8, HOXA7, ZNF562 | ZNF490 |
| 1:196585427 | rs313837380 | G | A | RELA, NFKB2, ZFP410, SOX1, ZNF619, ZBTB24 | FOXM1, ATF4 |
| 1:196603689 | rs14939226 | G | A | ZNF383, ZNF71, RBPJL, RBPJ, ZNF341, ZNF212, ZNF768, IKZF3 | IRF2, STAT2, NFATC2, POU2F1, ZNF582, IRF3, STAT1, PRDM6, IRF1, RFX4, PRDM1, RFX7, NFATC3 |
| 1:196603917 | rs14939230 | T | G | ZFP105, HOXB13, CPEB1, HOXA13, HOXC13, HOXC10, FOXC2, ZNF350, FOXC1, FOXL1, ZNF225 | FOXI1, ARID3A, POU5F1, NANOG |
| 1:196603944 | rs14939231 | C | T | ZNF891, ZNF224 | REST, ZNF675 |
| 1:196604150 | rs315994985 | C | A | MYOG, ASCL1, TCF3, MAFB, ZNF93, TCF12, MAFA, TFAP4, ZBTB18, ZBTB42, SMAD3, ZIC3, MYF5, BACH2, ZFP69, TWIST1, MYB, ZIC2, MSC, ASCL2, BHLHE22, TWIST2, TCF4, ZNF483 | MSGN1, PRDM5, ZNF558, HAND2 |

（To be continued on next page）

(Continued from previous page)

| rs | rsID | WT | MT | Specific transcription factor binding site before mutation | Specific transcription factor binding site after mutation |
|---|---|---|---|---|---|
| 1:196607410 | rs13998573 | C | T | ZFP410 | ZNF605, ETS1, SPDEF |
| 1:196607763 | rs317696844 | C | G | JUND, FOSL1 | |
| 2:85982181 | rs14945407 | T | A | ZNF345, ZBTB3, ZNF502 | ZNF74, ZNF621, NHLH1, T, ZNF135, SMAD5, RXRA |
| 2:103139584 | rs314080876 | T | G | OLIG2, MITF, HES6 | ZNF266, PAX6, TRP53, RBPJ, ZFX, LYL1, ZFP64, TFDP1, ZNF486, ZNF711 |
| 2:103140193 | rs80730241 | C | T | | SOX7, SOX21, SOX3, SOX17, SOX18, SOX11, SOX2, SRY, SOX8, SOX1, ZNF582, SOX9, SOX10 |
| 2:103141420 | rs80654245 | C | T | VENTX, YY1, HOMEZ, ZSCAN2 | ZFP260, BACH1 |
| 2:128794002 | rs734475775 | C | G | ZFP445, SOX17, SOX10 | TFCP2, AIRE, ZNF211, SOX14 |
| 2:128811387 | rs315917362 | G | A | NR1H4, HIC2, ZNF416, ZNF891, NANOG, ZNF431, ZNF449 | MAFG, SIX2, SIX4 |
| 3:3070317 | rs315618371 | T | C | SOX3, SOX9 | NR1H3, IRX3, POU3F1, ZNF565, IRX1, BATF |
| 3:31980122 | rs3386518642 | A | G | PPARG, ZFP532, ZBTB41, PLAG1, ZNF23, ZNF45, HOXB7 | RFX6, RFX7, RFX1, RFX3, PLAGL1, ZNF573, RFX4, RFX2, ZNF502, ZFP329 |
| 3:31980198 | rs318017131 | C | G | EN1, ZNF548 | RUNX1, RUNX2, ZNF708, ZNF502, ZNF382, MAFF, RUNX3 |
| 3:44253181 | rs314758575 | G | A | ZNF799 | NR2E3, VSX2, SIX6 |
| 3:44286804 | rs314559836 | C | T | HMX1, ZFP82 | BCL6, POU3F2, ZNF79, GATA5, FOXG1 |
| 3:44286842 | rs317987997 | G | C | ZNF620, ZNF274 | |
| 3:44309828 | rs316230214 | G | C | HMX2, ARNT, NKX2-4, HMX1, NKX2-6, SOX10, ZNF329 | ZNF442, ZNF214 |
| 3:44312747 | rs317042042 | C | T | NKX3-1, ZNF79 | POU3F2, FOXA3, ZNF418, FOXA1, TLX2 |
| 3:46102485 | rs315830313 | G | A | ZNF24, POU1F1, POU4F3 | ZFP784, HOXC12, NKX6-2, ZNF35, HOXC11, HOXC10, HOXA9, HOXA10, ZNF225, CDX2, HOXD12, CDX1, FOXO6, FOXO1, HOXD10, HLX, ZFP105, HOXD9, ZNF418 |
| 3:46104342 | rs317957463 | C | T | ZNF782 | ZNF667, SIX2 |
| 3:75849394 | rs314111651 | C | T | ZFP202, ZNF33A, INSM2, THAP1, PRDM16, ZNF257, ZNF792 | ZNF582, ZNF439 |

(To be continued on next page)

`

(Continued from previous page)

| rs | rsID | WT | MT | Specific transcription factor binding site before mutation | Specific transcription factor binding site after mutation |
|---|---|---|---|---|---|
| 4:13403229 | rs313026403 | C | T | ZNF398, PBX3, MYOD1 | POU3F2, POU3F1, POU3F4, POU1F1, POU6F1, HOXA2, LBX2, ISX, PRRX2, LMX1B, NKX6-2, MEOX2, NKX2-2, POU4F3, SHOX, LMX1A, LHX4, MEOX1, PDX1, DLX3, POU2F2, GSX1, ALX3, NKX3-2, BSX, POU2F3, EVX2, VSX2, NKX6-3, NKX6-1 |
| 4:13410951 | rs312919885 | T | C | PBX3, LHX2 | SPIB |
| 4:13412185 | rs14041540 | T | C | | IRF7 |
| 4:13412706 | rs316916496 | G | T | ZNF37A, FEZF1, E2F6, ZNF681, MAZ, ZBTB17, E2F3, ZFP14, CTCF, E2F7 | ZNF274, ZNF480, ZNF212 |
| 4:49898600 | rs314633821 | G | A | ZBTB41, ZIC3, ZBTB7B, ZNF701, ZNF774, INSM1, MAZ, ZNF587, ZFP770, ZFP82 | PPARA, PPARG, RARA, RXRA |
| 4:85072848 | rs316234116 | G | A | OSR2, SNAI2, ETS1 | |
| 4:85080308 | rs312880760 | A | G | | ZBTB18, PAX6, ZBTB42 |
| 4:85126506 | rs313768959 | T | G | ZNF419, ZNF257, ATF4, CEBPG, PAX6, HIC1, NFATC3, CEBPB | RBAK, ZNF566, ZNF681, ZNF132, JDP2, ZNF432, BACH2, BCL6, ELF3, FOSL2, ZNF530, ELF1, ETV1, ZNF18, SPIB, FOSB, XPA, ZNF770 |
| 4:85163257 | rs316272980 | T | C | | |
| 4:85174363 | rs315105277 | A | G | MAFG | |
| 4:85174958 | rs739153977 | G | A | TRP53 | POU5F1, POU2F3, POU5F1B, POU2F1, POU3F1, GATA3, GFI1, POU3F4, FOXA1, ZSCAN16, FOXA3, GFI1B |
| 4:85174966 | rs14416983 | A | G | SOX9, TRP53 | PAX6, PAX4, ZNF563, PAX5, PRDM14 |
| 4:85186151 | rs317436117 | A | G | ZNF506, NFIB, NFIA | TFAP2C, ZNF573, ZNF479, NFKB1, ZIC3, ZNF611, NFKB2, ZBTB7B, EGR1 |
| 4:85186566 | rs315254872 | G | A | ZNF200 | SOX10, SOX9, PRDM5, RFX5, ZNF432, ZNF680, PRDM9 |

(To be continued on next page)

`

（Continued from previous page）

| rs | rsID | WT | MT | Specific transcription factor binding site before mutation | Specific transcription factor binding site after mutation |
|---|---|---|---|---|---|
| 4:85186726 | rs312661958 | G | T | ZFP3, SP9, RFX5, EGR4, SP8, GLI3, SP1, EGR3, SP4, ZNF76, KLF15, ZBTB1, SP3, KLF13, KLF14, KLF5 | NKX2-2, TBX1, ZNF18, NKX3-1, RUNX2, ZFP1, EOMES |
| 4:85186875 | rs317783073 | C | T | KLF3, ZFP526, ZNF419, ZFP112, SOX15, NR1H4, RUNX2 | HOXD13, EBF4, PAX5, ZNF652, PPARD |
| 4:85186906 | rs316596701 | G | A | ZNF708 | BATF, OSR2, JUND, OSR1, ZBTB32, ZNF382, ELF3 |
| 4:85188198 | rs315646870 | G | A | PAX4 | EBF4 |
| 4:85206627 | rs314840589 | A | C | DMRT1 | PAX3, ZFP668 |
| 4:85215012 | rs314029489 | A | G | IRF5, ZNF334, ZNF442 | BLIMP-1 |
| 4:85218175 | rs317961268 | A | G | ZNF740, ZFP668, SP1, E2F7, ZBTB41, TBX3, ZFP740, PRDM9, KLF6, ZNF684 | PATZ1, KLF15 |
| 4:85382858 | rs733195637 | G | T | ZFP1, NR2E1, NR4A1 | ZNF580, NFATC4, SPI1, IRF4 |
| 4:89114648 | rs316478876 | A | C | GLI3, MYNN | NKX2-5, NKX3-1, NKX3-2, NKX2-3, HMX2, ZKSCAN7, SREBF1, NKX2-6, SREBF2, NKX2-1, ISL2, ISL1, NKX2-4, NKX2-8, NKX2-2, NKX2-9, NANOG, ZFP653 |
| 4:89116915 | rs317056444 | T | C | ZNF774, ZFP382 | RELA, ZNF708, ZNF341, ZNF41, PRDM4, ZNF684, ZNF620, ZNF84, FOXD3, RXRA |
| 4:89121169 | rs312458031 | A | G | ELK4, ZFP536, ELK3, ETV4, ZNF485, FOXJ3, ZNF16 | ZBTB24 |
| 4:89125298 | rs14417266 | C | G | ZNF714, ZNF611, RREB1, ZNF490, CTCF | ZNF93, ZNF69, ZNF322, ZNF157 |
| 5:15985047 | rs14417271 | C | A | EGR1 | ZIM2, ZNF331 |
| 5:22365615 | rs431846683 | T | G | ZNF33B, TEAD2, ZNF567, SREBF1, TEAD4, TEAD1 | ZNF529, ZFP39, PRDM9, ZFP668, BCL6, ZNF263, TBX20, NPAS4, ZNF189, ZNF132, RBPJ, SP5, PAX4, ZNF180, TBX3 |
| 5:28043501 | rs316576299 | A | G | YY2, ZFP668, IRF7, YY1, MZF1, NFATC2 | ZNF529, ZNF784, ZBTB7C, ZBTB7B, ZBTB7A |
| 5:56580820 | rs16087237 | C | T | | ZNF200, STAT6, T |
| 5:56593944 | rs16087254 | A | G | ZNF708, PITX1, ZFP740 | ZNF311, KLF3, PATZ1, ZFP579, NFKB2 |
| 5:56596059 | rs14228382 | T | G | SREBF1, ZFP513, NR1I3, TBX20, ZFP787, SREBF2, THRB | ZNF610, SP5, ZNF705G, IRF5, ZFP287, ZNF263 |
| 5:56596084 | rs314357717 | T | C | | IRX2, IRX5, TP73 |

(（To be continued on next page）

`

（Continued from previous page）

| rs | rsID | WT | MT | Specific transcription factor binding site before mutation | Specific transcription factor binding site after mutation |
|---|---|---|---|---|---|
| 5:56615492 | rs313700011 | T | G | PRDM5, ZNF502 | NFIX, NFIB, NFIC, FOXB1, ZFP46, ZNF506, KLF6, RFX1, ZNF264, ZNF157, AR, ZFP329, KLF5, BATF, ZBTB12, NFAT5 |
| 5:56616348 | rs738189398 | T | C | ZFP553, ZSCAN31, BCL11B | ZNF548, ZNF549, ZNF93, OSR2, ZBTB12, ZFP37, ZNF543, ZNF12, ZNF519, KLF3, ZNF490, TAL1, ZNF582, ZNF571 |
| 5:56618857 | rs314686498 | G | A | ATF6B, RHOX11, ZIC1, ZIC3, ZFP184, ZIC5, IRX5, ATF6, ZIC2, GLIS1 | PRDM5, ZKSCAN1, ZNF780A |
| 6:5075200 | rs16248915 | T | A | CDX2, EVX2, CDX1, HOXA10 | |
| 6:5075240 | rs16248917 | T | C | POU2F3, ZNF418, ZSCAN16 | ZFP786 |
| 6:5076732 | rs16263103 | C | A | ZNF274, RREB1 | ZKSCAN2, ZNF384, ZNF613, ZNF736, PAX4, RUNX2, ZNF22, ZNF805, RUNX1, MEF2A, TCF7, ZNF487 |
| 6:5078632 | rs313237810 | G | A | GFI1 | ZNF341, PGR, AR, ZNF614, NR3C1, ZSCAN9, RHOX11 |
| 6:5081150 | rs734297511 | A | C | | ZBTB5, ZKSCAN1 |
| 6:5084601 | rs733775780 | G | A | ZNF345, ZFP335 | ZNF85, TEAD2, TEAD1, ZNF614, TEAD3, TEAD4, FOXH1, TRP53, EBF4, ZNF778 |
| 6:5100033 | rs317193066 | A | G | ZNF300, PLAG1, ZNF780A, ZNF439 | ZBTB33, ZNF282, ZNF28, ZNF529, ZNF76, EGR2, ZNF267, ETS1 |
| 6:10310975 | rs735459861 | A | G | FOSL1, ZNF669, FOSL2, NR4A1 | |
| 7:23237809 | rs739305450 | G | A | HINFP | ZNF35, HNF4G, IRX2, ZNF564, ANHX |
| 7:23238115 | rs16302600 | A | G | NKX2-5, NKX2-4, RORA | ZNF281, ZNF260, KLF15, ZFP281, ZNF148, SP1, ZBTB17, ZFP316, SP2, SP4, KLF12, E2F7, ZNF157, ZSCAN5, KLF5, SP3, ZNF329, ZNF436, SP5, ZNF468, ZFP513, WT1 |
| 7:23238248 | rs13644638 | G | A | SMAD3 | ZNF85, OTX2, OTX1, PAX7, RHOX11 |
| 7:23238382 | rs15500309 | A | G | IRX5, SIX4, SIX2, ZNF454, SIX1, ZNF267 | |

(（To be continued on next page）

（Continued from previous page）

| rs | rsID | WT | MT | Specific transcription factor binding site before mutation | Specific transcription factor binding site after mutation |
|---|---|---|---|---|---|
| 7:23239760 | rs10722908 | C | T | PAX4, RFX5, PAX6 | HOXB13, CDX1, ZNF214, CDX4, HOXA13, ZNF799, HOXC13, ZNF287, HOXD13 |
| 7:23241529 | rs317910469 | T | C | ZNF3, ZNF768 | MYBL1, ESR2, MYB, MYBL2, ZNF324 |
| 7:23685239 | rs15500320 | A | G | HSFY2, ZFP787, HSFY1 | ZNF135, REST, RARG |
| 7:23691084 | rs315259024 | T | C | SIX4, SMAD4, ZBTB6, SIX1 | ESR2, ZNF454, RARG, ZNF692, ESRRA, ZNF550, RORA, ZNF322, ZNF12, AR |
| 7:23691185 | rs318238166 | C | T | JUN, JUND, FOS, FOSL2, FOSL1, JUNB, ATF3 | SOX1, ZNF250, ZNF24, ZNF586, ZNF713, ZNF184, POU4F3, FOXP3 |
| 7:23691531 | rs314239110 | C | T | ZNF302, XBP1, SPDEF, MAX, TRP53 | |
| 7:23705417 | rs15612774 | A | G | NR2E3, PRDM4 | ZNF436, ELF3, FEV, ZNF343 |
| 7:23706503 | rs315549948 | G | A | IRF3, XBP1, NFATC3 | SOX2, IRF7, NFAT5, SRY, SMAD4 |
| 7:23707501 | rs735858829 | A | G | RFX3, RFX2, NR2E1, CREB3L1, TBX1, ZNF558, POU5F1, MAFG, HES5 | ISL2, ISL1, ZSCAN10, RELA |
| 7:23713609 | rs313639521 | C | T | FOXA3 | REST, MAF, ZNF440, CTCF |
| 7:23715340 | rs14693158 | T | C | NFKB1, RELA, BLIMP-1, NFKB2, RELB, REL | THAP1, ZNF454, PATZ1, ZFP69B, ZFP513, ZNF468 |
| 7:23719007 | rs80768722 | A | G | ZNF212, ZNF84, FEZF1, ZNF354A, ZNF527, ZNF586 | ZNF35, ZNF440, ZNF383 |
| 7:23722085 | rs16691660 | T | C | AR, ZNF530, ZNF69, ZNF224, ZNF585A | ZNF791, NRF1, ZSCAN30, HNF4G, NR2F1, VEZF1, HNF4A, RUNX2, ZNF528 |
| 7:23729993 | rs16691659 | G | A | ZNF436, FOXA1, HNF4A, HNF4G | ZNF250, RXRA, VDR |
| 7:23732444 | rs14507727 | A | G | | PBX1, ZNF410, ZSCAN5, ZNF281 |
| 7:23732471 | rs315784018 | T | C | NFAT5, ZFP260, ZNF189, ZNF75A, THAP11 | PRRX2, ELF5, LHX4, NR2C2, ELF2, ISX, ELF1, ALX3, ELF3, EHF, VSX1 |
| 7:23732517 | rs316191687 | A | G | BCL6, ZFP382, BCL6B, EN1, NANOG | NEUROD1, ZNF2 |
| 7:23741246 | rs80587559 | C | T | NFATC1, ZNF565 | ZNF287, POU6F1 |
| 7:23743401 | rs739426901 | G | A | ZNF260, ZSCAN30, ZNF419, CREB3L1, NKX2-4 | NKX3-1, NKX3-2, ZFP787, ISL2, ISL1 |
| 7:23749916 | rs80730603 | A | C | ZFP319, ZNF496, MAF | ZNF454, ZKSCAN7, ZNF528, ZNF502 |
| 7:23752118 | rs318220524 | T | A | GRHL2, ZNF490, BCL11A | GFI1B, FLI1, ERG, HOXA9 |

（To be continued on next page）

`

（Continued from previous page）

| rs | rsID | WT | MT | Specific transcription factor binding site before mutation | Specific transcription factor binding site after mutation |
|---|---|---|---|---|---|
| 7:23753048 | rs739403822 | G | A | TBX2, KLF10, TBX5, EGR2, KLF11, ZFP1, RUNX2, EGR3, KLF17, TBX21, EGR1, FOXO3, KLF9, ZNF263, ZNF740, EGR4 | TEAD1, TEAD3 |
| 7:23754967 | rs80692083 | T | C | POU6F1 | TBX1, ZNF250, ZSCAN23 |
| 7:23755992 | rs315864220 | C | T | ZNF436, PRDM11, ZNF454, ZFP786 | ZNF792, RELB |
| 7:23756991 | rs314772558 | G | A | ETV5, NKX2-5, FOXP1, ZNF45 | DMRT3, ZNF184, ZNF684 |
| 7:23756996 | rs313942842 | A | G | NKX2-5, ZNF45, ZFP82 | MAZ, ZNF148, ZFP513, PATZ1, SREBF2, FOXO3, GTF3A, ZKSCAN5, ZNF534, ZNF548, ZNF880, ZNF770, ZNF580, ZFP641, ZNF281, ZNF740, KLF1, ZFP46, PRDM1, ZFP1, RXRA, PLAG1, E2F7, ZFP281, ZFP41, E2F6, ZNF785, PAX6 |
| 8:2866334 | rs317104670 | C | T | BHLHE40 | MTF1, FOXO6, ZNF605 |
| 10:7222733 | rs733428170 | T | A | BCL6B, ZNF34 | ZNF25, MAFF, MAFG, MAFK, MAFB, ZNF324 |
| 10:7224607 | rs80735721 | G | A | ZFP668, SOX2 | IRF1, IRF9, IRF2, IRF4, IRF8, STAT2, IRF5, JUND, IRF7, IRF3 |
| 11:15476382 | rs15690110 | A | G | ZNF74, ZNF582, ZFP184 | MAFA, NANOG, POU5F1, ZSCAN23 |
| 11:15477314 | rs16519176 | C | T | PBX1, ZNF619 | HOXA1 |
| 11:16237253 | rs731954564 | A | G | | GATA3, GATA2, ZNF737, GATA4, GATA5, GATA6, TRPS1 |
| 11:16311558 | rs316739697 | G | A | | POU1F1, POU4F3, POU4F2, POU4F1, POU3F3, POU2F2, POU3F1, HOXB8, PAX2, PAX1, PAX9 |
| 11:16311635 | rs318127048 | G | C | BHLHE41, HES2, SOHLH2, NR4A2, HEY1, HES7, TCFL5, FOSL1, NR4A1, TFEB, HIF1A, GMEB1 | CREB3L2, ATF6, MXI1, XBP1, SOX1, ATF6B, CREB3L4, CREB3, NKX2-1 |
| 11:16323284 | rs314518447 | A | G | ZBTB12, ZNF613, ZNF564, ZFP574, ZNF384, HSF5, HSF2, ZNF350, TRP53, ZEB1, HSF1, AHCTF1, ZBTB43, PTF1A, RHOX11 | EXPORT |
| 11:16348866 | rs14554663 | T | C | ZNF124, DDIT3, ATF4, CEBPG, BATF3, VSX2, MAF, T, CEBPB, DUXA, ESR1 | NFATC3, ZSCAN23, HNF4A |

（To be continued on next page）

`

（Continued from previous page）

| rs | rsID | WT | MT | Specific transcription factor binding site before mutation | Specific transcription factor binding site after mutation |
|---|---|---|---|---|---|
| 12:1727650 | rs15743050 | C | G | ZNF93, MAF, ZNF582, MAFG | ZFP319, FOXA1 |
| 12:11801847 | rs16527532 | A | G | SCRT2, SCRT1, IKZF1, SPDEF | ZNF90, ZSCAN31, ZNF250, PAX4, ZFP112, ZIC2, NEUROD2 |
| 12:11801904 | rs315866764 | T | C | TCF7 | CUX2, RARA, ZFP184 |
| 12:11801963 | rs312861187 | G | A | ZEB1 | GATA2, PRDM6, ZNF235, GATA3 |
| 12:11801979 | rs312491466 | A | G | ZNF570, FOXD2, SOX14 | TBX20 |
| 12:11802112 | rs312592149 | T | A | PBX1, PBX4, SOX8, ZSCAN4C, TCF7L2, GATA3, TCF4, LEF1, TCF3, TCF7L1, ZNF582 | ZNF287, ZFP1, ZNF492, SOX2, ZFP105, CEBPD, NKX2-5, ZNF235, ZNF225 |
| 12:11802772 | rs312973670 | G | A | NFATC1, ZNF69, ZNF799 | ZNF287 |
| 12:11802862 | rs315588466 | T | C | FEZF1, ZNF418, OLIG2, PTF1A, ZNF41, ZNF98, ZNF93, ZNF71 | PRDM4, BCL6, ZNF85 |
| 12:11802865 | rs16530658 | T | C | ZSCAN2, ZNF302, SPIC, MEF2A | ZNF549, NR4A1, ZNF84, KLF15, ZNF341, ZNF492, ZNF394, ZNF774, NR2E3, ZNF432, SOX4 |
| 12:11804201 | rs314184780 | A | G | ZNF134, ASCL2, NHLH2, ZNF273, TBX20 | NKX2-5, NKX2-8, NKX2-3, NKX2-2, NKX2-4, KLF14, NKX2-6, NKX2-9, ZBTB24, SP2, CTCF, SP5, TAL1, ZNF543, SNAI1 |
| 12:11804663 | rs15776234 | A | G | FOXP1, SOX10, TCF7, SOX3, SOX4, SOX2, NANOG, SOX11, SRY, FOXJ1, LEF1, ZNF98, FOXC1, ZFP105, SOX6, FOXO4, FOXD3, FOXO1, ZSCAN5C, FOXK1, FOXP2, ZNF354A, ZNF384, FOXJ3, FOXA1, ZNF879 | BCL11A, IRF2, IRF1, STAT2, ZNF71, STAT1, PRDM1, ELF3 |
| 12:11804863 | rs739635299 | C | A | RUNX3, RUNX2, ZBTB18, NHLH1, ZNF284, CBFB | DMRTB1, IRF6, IRF4, IRF8, RELA, IRF9, ZBED2, SPDEF, IRF2, SPI1 |
| 12:19780775 | rs316811946 | C | T | ZNF85 | RARA, RXRA, NR5A2 |
| 12:19783140 | rs316188605 | A | C | ZNF331, ZFP128 | ZNF558, ZSCAN4, FOXG1, ZNF778, ZSCAN1, KLF9, EGR2, FOXO3, GCM1, EGR1, RUNX2, ZNF816, ZNF821, CBFB, FOXO6, KLF17, TBX5, ZNF524 |

（（To be continued on next page）

`

（Continued from previous page）

| rs | rsID | WT | MT | Specific transcription factor binding site before mutation | Specific transcription factor binding site after mutation |
|---|---|---|---|---|---|
| 12:19784461 | rs314985840 | A | G | ZNF69, ZNF419, ZNF331, ZNF596 | ZNF701, ZSCAN31, ZNF16, SIX5, ZNF860, GTF3A, ZNF132, LEF1, RELB, SP4, TCF4 |
| 12:19786586 | rs793931460 | C | T | TBX20, SMAD3, ZNF821, ZNF7, TAL1, TBX21, MEIS1, ZFP553, ZNF554, PKNOX2 | FOXE1, FOXD2, FOXL1, FOXC1, ZFP770, FOXM1, ZNF432, FOXD3 |
| 12:19801364 | rs739919594 | G | T | ZBTB42, HES7, BHLHE41, ATOH1, MAX, HEY2, ARNT, MYRF, ASCL1, TFE3, HES1, HES5, TCF21, NHLH2, HIC1, NEUROD1, HES2, MSC, MXI1, OLIG3, HEY1, TP63, MYC, ASCL2, ZBTB43, TFEB, MLX, TCFL5, MYCN, TP73, ZNF76, ZNF563, NFIX, TAL1 | TBX2, ZBTB12, PRDM5, ZNF619 |
| 14:1238758 | rs316991790 | A | G | BBX, HBP1, SOX10 | ZNF552 |
| 14:2508316 | rs313792827 | C | A | DUX4, ZNF140, ZNF3 | ARID5A, FOXB1, FOXA2, SOX2, FOXC1, FOXD3, FOXC2 |
| 14:14628939 | rs312457584 | C | A | ZNF776, ZNF527, ZNF582 | REST, IRF6, CUX1, CUX2 |
| 18:7916856 | rs313224745 | A | G | NFE2L2, ZBTB24 | TEF, CREB3L1, ZNF311, ATF6, ATF6B, CREB3L2, XBP1, CREB3, HLF, CREB3L4, BATF3, NFIL3, GMEB2, RFX3 |
| 18:9947166 | rs740514701 | T | C | ZNF121, ZSCAN31, ZFP3, TFCP2 | TBX5, PAX6, SP2, ZNF684, ZNF792, TBX20, SP5, ZNF816 |
| 18:9950906 | rs316894769 | C | A | ZNF776, MTF1, ZSCAN4C, E2F2 | ZNF382, TBX20 |
| 18:9959147 | rs15864493 | C | G | ZNF225, ZFP182, LBX1, IRF3, BCL6, ZNF586, TCF3, ZFP112 | FOXC1, SPDEF, MGA, PBX1, HOXA2, T, TBX2, HOXB7, TBX5 |
| 18:9960650 | rs314090469 | A | G | POU2F1, ELK3, POU5F1, POU2F2, POU3F1, FOXE1, FEZF1, FOXB1, FOXD2, POU2F3, FOXJ3, FOXK1, FOXG1, ZNF716 | ELF1, SPI1, ELF3, NR2C2, ELF2, GABPA, BCL6B, TBX1 |
| 19:6505623 | rs14617318 | A | G | AIRE | SALL2, ZNF41, ZNF524, HES6, MAX, SOHLH2 |
| 22:461024 | rs14617319 | C | T | BARHL1, ZNF296, BARHL2, VAX2, RARB, ZNF133, NEUROG2 | ZNF35, TFAP4, PDX1, ZNF302, HOXB7, HOXC9, OLIG3 |
| 27:3796945 | rs313296176 | G | A | ZNF213 | ZNF114 |

（To be continued on next page）

`

（Continued from previous page）

| rs | rsID | WT | MT | Specific transcription factor binding site before mutation | Specific transcription factor binding site after mutation |
|---|---|---|---|---|---|
| Z:30960219 | rs313456709 | T | C | AR, FOXD3, FOXC2, FOXI1, ZFP105, FOXD2, FOXJ2, CTCF, FOXL1, FOXA1, ZNF182, ZNF805, FOXA3, PRDM6, FOXG1, FOXR2, FOXM1, FOXR1 | SOX12, SOX4, NANOG |
| Z:30982495 | rs730934738 | C | T | ZNF135 | SOX18, POU4F3, SOX13, ZNF140, POU4F2, POU3F1, HESX1, RARA, RARB, SOX14 |
| Z:31467999 | rs312449117 | C | T | ZNF329, BCL6B, ZNF140, ZNF614 | |
| Z:31468208 | rs738431855 | A | G | ZFP14, ZNF527, SIX2, ZNF341, ZNF680, SIX1, ZKSCAN2, NFKB2 | ZNF415, ZBTB6, OSR2, ZNF708, NHLH1, ZNF530, PTF1A, ZIM2, BHLHA15, ZNF93, OLIG2, ZFP69B, SP1, ZNF71, ZNF519 |
| Z:31468803 | rs3388220694 | T | C | FOXA1 | ZNF558, SCRT1, ZKSCAN7, SCRT2 |
| Z:31469397 | rs317018855 | C | T | SIX4, GATA4, GATA3, GATA5, GATA6, GATA1 | ZFP367, FOXR1, ZFP654 |
| Z:31470828 | rs314490595 | T | C | ZNF483, LYL1, SIX5, E2F6, E2F7, PTF1A, TFDP1, HES7, ZNF69, ZFP46, E2F3, TCF4, ZNF398 | ZNF148, ZBTB7A, ZNF460, ZNF543, CTCFL, ZNF180, ZNF100, EGR1, PLAG1, ZBTB17, ZNF571, PRDM5, ZNF740, KLF9, ZNF260, KLF3, SALL4, KLF12, PEG3, ZBTB7B, ERG, ZNF320, KLF16, ZFP513, ZNF197, ZNF707, ZNF684 |
| Z:31471141 | rs313488012 | C | T | | DMRT1, DMRTB1, NR2F1 |
| Z:31471295 | rs313387429 | T | C | ZNF317, YY1 | ZNF154, IKZF3, RBPJ, BLIMP-1, RBPJL, CEBPE, E2F3 |
| Z:31474541 | rs735129014 | T | G | ZNF484, NFATC1, ELF2, ZNF880, ZNF224, ETV4, ZNF212, DMRT1, ZNF480 | IRX5, BCL6B, PAX5 |
| Z:31474926 | rs733539751 | T | C | ZNF398, ZNF530, NHLH1 | TAL1, GATA1 |
| Z:31475239 | rs16776397 | T | C | RFX3 | SREBF1, ZNF549, SREBF2 |
| Z:31475464 | rs741291342 | A | T | FOXP3 | |
| Z:31476489 | rs316071695 | G | T | RBPJ, ZNF484, ZNF701, ZNF394, RELA | NFATC3, NFAT5, NFATC4, ZNF189 |
| Z:31478846 | rs733148472 | A | C | ZNF12, HOXD3, TEAD4, HOXB5 | LHX1, GATA4, ZFP287 |

（To be continued on next page）

`

（Continued from previous page）

| rs | rsID | WT | MT | Specific transcription factor binding site before mutation | Specific transcription factor binding site after mutation |
|---|---|---|---|---|---|
| Z:31481217 | rs736318169 | T | A | SOX10, ZNF337, PAX3, MSGN1, TLX3, ZFP322A, SOX3, SOX13 | ZFP184, FOXP1 |
| Z:31492476 | rs314955625 | A | T | IRF6, NFIL3 | |
| Z:31492800 | rs313371449 | A | G | FOXA1, HOXB13, HMG20B, HOXA13, BARX1 | GATA1, LHX9 |
| Z:31494181 | rs14778403 | A | G | ZNF181, TRP53, POU2F2 | CTCF, ZNF331, ZBTB43, ZBTB18, TWIST1, NHLH2 |
| Z:31494359 | rs312440942 | T | C | MESP2, MSGN1, NKX1-2, SOX5, TLX3, SOX6, ZNF317, SOX13 | FOXD3, FOXA1, FOXJ1 |
| Z:31501598 | rs740312969 | T | C | HOXA9, PAX5 | ZNF550 |

`

**Table S3.** Comparison of the difference of affinity of transcription factors before and after mutation

| rs | rsID | WT | MT | Specific transcription factors binding before mutation | Specific transcription factors binding after mutation |
|---|---|---|---|---|---|
| 1:27173461 | rs15216781 | C | T | OLF1, BRACH, MYCMAX, USF, GR, NMYC, TAL1ALPHAE47, E47 | HOX13, S8, OCT1, VMYB, XBP1, SREBP1 |
| 1:27184061 | rs315145701 | T | C | AHRARNT, TATA | HFH1, ELK1, YY1 |
| 1:27184104 | rs314397809 | C | T | STAT, TATA | OLF1, RFX1, OCT1, ZID, MEF2, EVI1, CEBPB, CEBPA, SOX5 |
| 1:27184143 | rs13842215 | T | C | POLY, CEBP, NFY, CAP | COMP1, ZID, GC, SP1, STAF, RREB1, GATA2, GATA3 |
| 1:27184545 | rs735711891 | C | T | AHRARNT, GC, VMAF, NFE2, CAP | GR, CP2, CEBPB |
| 1:27196512 | rs13842248 | C | T | TAXCREB, RREB1, NFKAPPAB50, AP2, STAF, E2F, AHR | HOX13, OCT1, CAP |
| 1:27196619 | rs317694843 | T | C | | CAP, MIF1, AP2, EGR1, NGFIC |
| 1:27237136 | rs315047313 | C | T | AP4, LYF1, ATF, CP2 | MYOGNF1, CDP, AHRARNT, PBX1, NFY, SRY, GC |
| 1:27240573 | rs317433662 | T | G | TST1, S8, AP1, NFE2, CDPCR1, USF | CHOP, E47, NGFIC, VMYB |
| 1:27243212 | rs317015848 | T | G | TH1E47, TATA, EVI1, ARP1, CAAT | OLF1, MYOD, NRF2 |
| 1:27283069 | rs314576853 | A | T | COMP1, AHRARNT, ISRE, IRF1, VMYB, ZID | CEBPA, CHOP, AP1, GATA2, LMO2COM |
| 1:27406542 | rs312848604 | G | A | AHRARNT, CETS1P54, E2F | MYOGNF1, CP2, STAF |
| 1:60619217 | rs317224719 | C | T | PAX2, STAF, CMYB, GATA1 | ATF, USF |
| 1:83781180 | rs314346696 | T | C | RREB1, GR, GRE, IRF2, CAAT, HNF3B, EVI1, HNF4, XFD3 | CETS1P54, R, MYB |
| 1:176289110 | rs313999630 | A | C | GFI1, CREB, CMYB, GR | AHRARNT, P300, NFKAPPAB65, CREL, ZID, SRY, GC, R |

（To be continued on next page）

`

（Continued from previous page）

| rs | rsID | WT | MT | Specific transcription factors binding before mutation | Specific transcription factors binding after mutation |
|---|---|---|---|---|---|
| 1:176289152 | rs317629975 | A | C | XFD3, HNF3B, POLY, TAL1BETAE47, TAL1BETAITF2, VMAF | STAT3, NFKB, GATA, NFKAPPAB50, NFKAPPAB65, NFKAPPAB, HSF1, HSF2, GATA2, E2F, IK2, EVI1, LYF1, SEF1, VMYB |
| 1:176299324 | rs14922464 | A | T | HOX13, S8, VMYB, SRY, STAT | CDPCR3, ISRE, POLY, TH1E47 |
| 1:176306216 | rs15512540 | C | T | E2F, ZID, VMYB | ER, ELK1, CETS1P54, NRF2, LYF1 |
| 1:176307593 | rs315526298 | G | A | CETS1P54, IK1, IK2, CDPCR1, CDPCR3HD | VMYB, ELK1, SRY, CDPCR3, GATA1, EVI1 |
| 1:176315868 | rs313356458 | C | T | CDPCR1, YY1, E47, TAL1BETAITF2, GATA3, CDPCR3HD, MYOD | NRSF, RORA1, CREBP1CJUN, EVI1 |
| 1:187090099 | rs738182074 | C | T | ZID, GATA2, GATA3, MYOD, ARNT, MYCMAX, MAX, USF, NMYC, SREBP1 | CETS1P54, ELK1, OCT1, E2F, MEF2 |
| 1:187091008 | rs316526526 | C | G | MEF2, PAX2, MYCMAX, MAX, USF, NMYC, P53, MYB | CREB, CETS1P54, GR |
| 1:196509446 | rs316211752 | C | T | E2F, MIF1, E2 | STAT1, NRF2, E4BP4, HLF, VMYB |
| 1:196522180 | rs313962930 | G | A | YY1, RFX1, HSF2, VMYB, CETS1P54, CEBP, HNF4 | HNF1, IRF2, TATA |
| 1:196522212 | rs734842753 | G | A | VMYB, NFY, SP1 | COMP1, ER, XFD3, ATF, CREB, AP1, USF, DELTAEF1, SREBP1, LMO2COM, ELK1, TST1, OCT1 |
| 1:196530963 | rs16004403 | A | G | E2F, OCT1, YY1, PAX2, E47, TAL1BETAE47, TAL1ALPHAE47, TAL1BETAITF2, SRF, SRY, NMYC, P53, TATA | HSF1, HSF2, CEBP |
| 1:196549561 | rs317881329 | C | G | GR, SP1, LYF1, BARBIE | CREB |
| 1:196557882 | rs14939151 | A | G | OLF1 | STAT1, STAT3, E2F, MZF1, LYF1, STAT, AP1 |

（To be continued on next page）

`

（Continued from previous page）

| rs | rsID | WT | MT | Specific transcription factors binding before mutation | Specific transcription factors binding after mutation |
|---|---|---|---|---|---|
| 1:196557890 | rs316655562 | G | C | AHR | RREB1, AP4, OCT1, IK3 |
| 1:196573425 | rs316889139 | T | G | SRF, SRY | SEF1, GC, GATA1, USF, PADS, VBP |
| 1:196573490 | rs314830954 | C | T | TH1E47, GATA1, GATA2, HSF1, HSF2, USF, AP4, CP2 | VMYB |
| 1:196574056 | rs14939165 | A | T | CEBPA, LYF1, HSF2, GATA, PADS | ARP1, ER, GR, YY1, IK3, NFKAPPAB50, NFKAPPAB65, NFKAPPAB, T3R, VJUN, XBP1, ATF, AP1FJ, AP1, TAXCREB, CREB, CREBP1CJUN, COUP |
| 1:196580813 | rs314968551 | C | T | SP1, LMO2COM, MYOD, AP2, E47 | GRE, MYOGNF1 |
| 1:196580993 | rs1058415596 | C | T | AP4, AP2, VMYB | CAAT, COMP1, SOX5, OCT1 |
| 1:196581449 | rs313415787 | G | A | ARNT, CP2, MYCMAX, USF, NMYC | MYOGNF1, COMP1, AP1, OCT1, OCT |
| 1:196582730 | rs1059181375 | A | G | MEF2, POLY, HNF1, CAAT | IRF1, IRF2, CDPCR3, GATA3, PAX5, CREB |
| 1:196585427 | rs313837380 | G | A | COMP1, AP2, OCT1, IK1, LYF1, SP1, NFKB, NFKAPPAB50, NFKAPPAB65, NFKAPPAB, CDPCR3HD | CEBPB, CEBPA, E4BP4, OCT, CETS1P54 |
| 1:196603689 | rs14939226 | G | A | GATA2, IK1, IK2, AHRARNT, E2F | YY1, P53, OCT, CP2, SRY |
| 1:196603917 | rs14939230 | T | G | E2, XFD1, XFD2, HFH1, HFH2, TST1 | CAAT, GFI1, CLOX, PBX1, NFY, MYOGNF1, COMP1, EVI1, CEBPA, P53, CEBPB, CEBP, STAF |
| 1:196603944 | rs14939231 | C | T | E2, CMYB, R, MYB, VMYB | IRF2, OCT1, GATA1, CDP, HSF2, CEBP, CEBPB |
| 1:196604150 | rs315994985 | C | A | VMAF, AP1FJ, NFE2, RFX1, PAX5 | YY1, TAL1BETAE47, GATA1, GATA3, CEBPB |
| 1:196607410 | rs13998573 | C | T | SP1, IK2, VMYB, MYB, CAAT, ATF, CEBPB | YY1, CETS1P54, CAP, HNF3B, CREB |

（To be continued on next page）

`

（Continued from previous page）

| rs | rsID | WT | MT | Specific transcription factors binding before mutation | Specific transcription factors binding after mutation |
|---|---|---|---|---|---|
| 1:196607763 | rs317696844 | C | G | OCT1, CREBP1, E4BP4, VBP, HLF | HOX13, XBP1, TAXCREB, GFI1 |
| 2:85982181 | rs14945407 | T | A | NKX25, CAP | HEN1, GC |
| 2:103139584 | rs314080876 | T | G | E2, MIF1, NRF2, ARNT, MAX, USF, NMYC, SREBP1, SP1 | IK2 |
| 2:103140193 | rs80730241 | C | T | RFX1, ATF, GATA1, GATA2, USF | PAX5, SRY, P53, SOX5 |
| 2:103141420 | rs80654245 | C | T | TST1, E2, ATF, T3R | AP1, CEBPB, ZID |
| 2:128794002 | rs734475775 | C | G | YY1, SP1 | CAP |
| 2:128811387 | rs315917362 | G | A | STAF, CP2 | AHRARNT, GC |
| 3:3070317 | rs315618371 | T | C | SRY, SOX5, CAP, GC | P300, PAX2, SEF1, STAF, NRF2 |
| 3:31980122 | rs3386518642 | A | G | PAX5, RREB1, SRY, SOX5, CDP, MYOD, TH1E47 | STAT |
| 3:31980198 | rs318017131 | C | G | E47, PAX5, TH1E47, ZID, CEBPB, NF1, CP2 | VMAF, AP4, USF, T3R, PADS |
| 3:44253181 | rs314758575 | G | A | CDPCR3, SRF, MYOD, E2, VMYB, CLOX, CDPCR1, E2F, GC | ELK1, TH1E47, CAP, XFD3 |
| 3:44286804 | rs314559836 | C | T | P53 | GATA1, GATA2, GATA3, CEBPB, CEBPA, CEBP, OCT |
| 3:44286842 | rs317987997 | G | C | VMAF, CREB, HNF1 | ER, CAP, OCT1 |
| 3:44309828 | rs316230214 | G | C | AP4, MAX, USF, MYOD, NMYC, MYCMAX, SREBP1, LMO2COM, NKX25, AP1, CREBP1, CAAT, HFH1, CEBP | CREB, CAP |
| 3:44312747 | rs317042042 | C | T | TAXCREB, HSF2, BRN2, CREBP1, TATA, NKX25 | GFI1, HNF1, VMYB, GATA3, XFD3, CEBPB, CEBPA, CEBP, PBX1 |
| 3:46102485 | rs315830313 | G | A | HOX13, IRF2, PAX6, PAX2, TST1 | PBX1, XFD2, GATA1, HFH1, HNF3B, TATA, NKX25 |

（To be continued on next page）

`

(Continued from previous page)

| rs | rsID | WT | MT | Specific transcription factors binding before mutation | Specific transcription factors binding after mutation |
|---|---|---|---|---|---|
| 3:46104342 | rs317957463 | C | T | HEN1, CAAT, GATA1 | YY1, OCT1, EVI1, SRY, T3R, TATA, CREBP1CJUN |
| 3:75849394 | rs314111651 | C | T | AHRARNT, ER, GC, OLF1, USF, ATF | |
| 4:13403229 | rs313026403 | C | T | LYF1, AP1 | SRF, TST1, TATA, NKX25, POLY |
| 4:13410951 | rs312919885 | T | C | EVI1, S8, CEBP, CAP | NFKB, ELK1, CETS1P54, IK1, IK2, CDPCR1 |
| 4:13412185 | rs14041540 | T | C | ELK1 | ZID, SP1, E2F, SEF1 |
| 4:13412706 | rs316916496 | G | T | TATA, LYF1, MZF1, POLY, GATA1, ISRE | SRF, P300, SREBP1, EVI1 |
| 4:49898600 | rs314633821 | G | A | GATA1, HSF1, HSF2, AP2 | PPARA, POLY, COUP, HFH1, ARP1 |
| 4:85072848 | rs316234116 | G | A | NFKAPPAB, HSF1 | P300, GC, NFKAPPAB65, E47 |
| 4:85080308 | rs312880760 | A | G | SRF | PAX5, ARP1, AP2, E47, STAF, MYOGNF1, DELTAEF1, ELK1, GATA2 |
| 4:85126506 | rs313768959 | T | G | MYB, CEBPB, CEBPA, CREBP1 | PAX5, P300, ZID, HSF1, AP1FJ |
| 4:85163257 | rs316272980 | T | C | GFI1, XFD3, SRY, MEF2, CEBPA, CEBP | CP2, HSF2, GC, CEBPB |
| 4:85174363 | rs315105277 | A | G | HNF1, NFY, CAP, CETS1P54, CEBP | GR, IRF2, E2F |
| 4:85174958 | rs739153977 | G | A | | E47, PAX6, AHRARNT, CEBP, AHR, GFI1, OCT, CHOP, GATA1, GATA2, GATA3, CEBPB, SRF |
| 4:85174966 | rs14416983 | A | G | OCT1 | PAX6, YY1, NMYC, NGFIC, CDPCR1, CDPCR3HD, SEF1, RORA1 |
| 4:85186151 | rs317436117 | A | G | NF1, RFX1, HSF2 | OLF1, NGFIC, CETS1P54, AP2, SP1, P300, NFKAPPAB50, CREL, NFKAPPAB |
| 4:85186566 | rs315254872 | G | A | VMYB, NFY, TAXCREB | GR, PAX5, YY1, ARP1, CDPCR3, NF1, AP4 |

(To be continued on next page)

(Continued from previous page)

| rs | rsID | WT | MT | Specific transcription factors binding before mutation | Specific transcription factors binding after mutation |
|---|---|---|---|---|---|
| 4:85186726 | rs312661958 | G | T | CP2, EGR1, SP1, TAXCREB, R | MZF1, ER |
| 4:85186875 | rs317783073 | C | T | ARP1, AHRARNT, PADS, CREB, GC, SP1, OCT1 | COUP, GATA2, PAX6, CDPCR3, CAAT |
| 4:85186906 | rs316596701 | G | A | NFE2, GC, SP1, P300 | MYOGNF1, CEBP, BRN2, CEBPB, CEBPA, E4BP4, TH1E47, VBP, GR, HSF2 |
| 4:85188198 | rs315646870 | G | A | CREB, CAP, VMAF, TST1 | TATA, CDPCR3HD |
| 4:85206627 | rs314840589 | A | C | COMP1, CMYB, SOX5, SRY, VMYB, MYB | NFY, XBP1, ARNT, MYCMAX, MAX, USF, NMYC, P53, SP1, TST1 |
| 4:85215012 | rs314029489 | A | G | | CEBP, RREB1, SP1, AP2, LYF1, E2F, STAT |
| 4:85218175 | rs317961268 | A | G | HOX13, EGR1, NGFIC, OLF1, NFY | LMO2COM, AP4, VMYB, COMP1 |
| 4:85382858 | rs733195637 | G | T | GFI1, E2, SREBP1, ATF, HNF4, CREBP1CJUN, HSF1, HSF2, P53 | NRF2 |
| 4:89114648 | rs316478876 | A | C | MYOGNF1, NF1, AHRARNT, AHR, CMYB, DELTAEF1, VMYB, E2, OCT1 | TAXCREB, P300, PADS |
| 4:89116915 | rs317056444 | T | C | PAX5, P53 | MYOGNF1, VMAF, HSF1, HSF2, GR, CMYB |
| 4:89121169 | rs312458031 | A | G | XFD1, XBP1, ATF, CDXA, VBP, CREBP1CJUN | P300, AHRARNT, OCT, T3R, NFKAPPAB50 |
| 4:89125298 | rs14417266 | C | G | SEF1, COMP1, IK3, CAP | AHRARNT, HNF3B, P53, MYOGNF1, HNF4, ARNT, POLY, MYCMAX, CHOP |
| 5:15985047 | rs14417271 | C | A | AHR, ER, EGR1, NGFIC, EGR2 | ARP1, BARBIE |
| 5:22365615 | rs431846683 | T | G | IK1, NMYC, MYCMAX, USF, T3R, CEBP, XFD3, PADS | STAF, TAL1ALPHAE47, E47, EGR1, NGFIC, EGR2, DELTAEF1, P300, ZID, SP1, TAXCREB |

(To be continued on next page)

`

(Continued from previous page)

| rs | rsID | WT | MT | Specific transcription factors binding before mutation | Specific transcription factors binding after mutation |
|---|---|---|---|---|---|
| 5:28043501 | rs316576299 | A | G | CP2, YY1, OCT1, CAP, OCT, ISRE | VMYB, MYB, IRF1, IRF2, IK2 |
| 5:56580820 | rs16087237 | C | T | GATA1, GATA2, GATA3, LMO2COM | HOX13, OCT1, S8, SRY, CEBPB, CEBP, GR |
| 5:56593944 | rs16087254 | A | G | MIF1, HNF4, OCT1, P53, R, CREL | AHRARNT, IRF1, IRF2, CP2 |
| 5:56596059 | rs14228382 | T | G | NFKB, HSF1, HSF2 | PAX5, AP4, GC, PADS, SP1, MZF1, CREB, T3R |
| 5:56596084 | rs314357717 | T | C | | HOX13, AP4, SRY, MYOD, NMYC, MYCMAX, SREBP1 |
| 5:56615492 | rs313700011 | T | G | COMP1, CEBPB, CEBPA | CMYB, PAX5, GATA2, P53, NF1, NFE2, E47 |
| 5:56616348 | rs738189398 | T | C | | YY1, LMO2COM, AP4, MYOD, CAP |
| 5:56618857 | rs314686498 | G | A | POLY, E2, VMYB | E2F, OCT1, XFD3 |
| 6:5075200 | rs16248915 | T | A | T3R, VMYB, ARP1, CAAT, AHRARNT | GC, CDPCR3, LMO2COM |
| 6:5075240 | rs16248917 | T | C | MYCMAX, GATA1, CAP | GC, SP1, AP4, MZF1, LMO2COM, CDPCR3 |
| 6:5076732 | rs16263103 | C | A | GC | MYOGNF1, ATF, CREBP1CJUN |
| 6:5078632 | rs313237810 | G | A | GFI1 | PAX5, GATA, GR, GRE, MYOGNF1 |
| 6:5081150 | rs734297511 | A | C | PAX5, PAX2, SRY, ARP1 | AHR, CMYB, P300, AP4, VMYB, CP2 |
| 6:5084601 | rs733775780 | G | A | RFX1, AHRARNT, AP1 | VJUN, CEBP |
| 6:5100033 | rs317193066 | A | G | TAXCREB | NGFIC, SEF1 |
| 6:10310975 | rs735459861 | A | G | VJUN, RORA1, T3R, PAX5, AP1FJ, AP1, E2, NFE2, CREB, CDP, CLOX, CDPCR1, CAP | ATF |
| 7:23237809 | rs739305450 | G | A | AHRARNT, AP1FJ, AP1 | COUP, YY1, MEF2, TATA, OCT |
| 7:23238115 | rs16302600 | A | G | RORA1 | PAX5, AP4, CREB, RREB1, LYF1, SP1 |
| 7:23238248 | rs13644638 | G | A | GC, SP1, TH1E47, PADS | CEBPB, CEBPA, SREBP1, HLF, CHOP, GATA1, OCT1, GATA2, GATA3, P53 |

(To be continued on next page)

`

（Continued from previous page）

| rs | rsID | WT | MT | Specific transcription factors binding before mutation | Specific transcription factors binding after mutation |
|---|---|---|---|---|---|
| 7:23238382 | rs15500309 | A | G | YY1, ARP1 | AHRARNT, E47, ARNT, MYCMAX, MAX, USF, PADS |
| 7:23239760 | rs10722908 | C | T | PAX5, OCT1, CHOP, CEBPB, CEBPA, ARP1, CAP, MIF1, E4BP4, EVI1, AP1 | POLY, YY1, MEF2, SRF, E2F |
| 7:23241529 | rs317910469 | T | C | ELK1, TAL1ALPHAE47, TAL1BETAITF2, GATA1, SRY, XFD3 | GR, GRE, COMP1, T3R |
| 7:23685239 | rs15500320 | A | G | CAP | CETS1P54, HSF1 |
| 7:23691084 | rs315259024 | T | C | NF1, AP1, MYOD | YY1, ER, GRE, P53, CP2, RORA2 |
| 7:23691185 | rs318238166 | C | T | GC, ISRE, STAF, MZF1, NRF2 | CAAT, GFI1, S8, CAP, HNF1, CDPCR3HD |
| 7:23691531 | rs314239110 | C | T | ARNT, ATF, MAX, NMYC, SREBP1, VBP, CREB, CREBP1, P53 | RREB1, CEBP, SRY, CETS1P54, IK3 |
| 7:23705417 | rs15612774 | A | G | S8 | CREB, CREBP1, COMP1 |
| 7:23706503 | rs315549948 | G | A | CHOP, XBP1, CREBP1CJUN, MYB, RFX1, MEF2 | MYOD, NFKB, GC, NFKAPPAB, OCT, CEBPB, CAP |
| 7:23707501 | rs735858829 | A | G | CEBP, IRF1, IRF2, TH1E47, GC | CHOP |
| 7:23713609 | rs313639521 | C | T | COMP1, GATA1, GR, ARNT, R, NMYC, AHRARNT | NRSF, STAF, MYOD, GATA2, P53 |
| 7:23715340 | rs14693158 | T | C | TAXCREB, COMP1, CREL, GATA1, GATA2, HSF1, HSF2 | RFX1, ARP1, MYB, GC, SP1 |
| 7:23719007 | rs80768722 | A | G | YY1, GR | CAAT, AP4, GATA3, GC |
| 7:23722085 | rs16691660 | T | C | CAAT, GR, STAF, SP1 | PAX5, NFKB, NFE2, COUP |
| 7:23729993 | rs16691659 | G | A | OCT1, YY1, TH1E47, GATA1, P300 | GR, HFH1, AP4, MYB, USF |
| 7:23732444 | rs14507727 | A | G | SRY, CEBPB, TAL1BETAE47, TAL1ALPHAE47, TAL1BETAITF2, LYF1, USF | GC, SP1, GATA1, GATA2, CDPCR1, CDPCR3HD, RREB1, SOX5 |

（To be continued on next page）

`

（Continued from previous page）

| rs | rsID | WT | MT | Specific transcription factors binding before mutation | Specific transcription factors binding after mutation |
|---|---|---|---|---|---|
| 7:23732471 | rs315784018 | T | C | ARNT, XBP1, RFX1, USF, MYCMAX, DELTAEF1 | HOX13, PAX6, PAX2, MYB, CETS1P54 |
| 7:23732517 | rs316191687 | A | G | COMP1, CEBPB, CEBPA, CETS1P54, IK1, IK2, CREBP1 | AP4, TAL1ALPHAE47, TAL1BETAITF2, DELTAEF1, STAT, AHRARNT, TAXCREB, MYOD, GC, GATA1, GATA2, GATA3, HSF1, HSF2 |
| 7:23741246 | rs80587559 | C | T | ELK1, CREL, HSF1, HSF2, CETS1P54, R, YY1, E4BP4 | PAX2, CDPCR1, OCT, SRF, EVI1, GATA, STAT |
| 7:23743401 | rs739426901 | G | A | CMYB, MYCMAX, MAX, USF, NMYC, LMO2COM, MYOD, CP2, LYF1 | |
| 7:23749916 | rs80730603 | A | C | OCT1, HNF1, NFKAPPAB65, IRF1, NKX25 | TATA, P300, ARP1, CAP |
| 7:23752118 | rs318220524 | T | A | NFY, P53, VMYB, MYB | GFI1, T3R, TAL1BETAE47, TAL1ALPHAE47, TAL1BETAITF2, RORA2, EVI1, GATA2, CDPCR3, ELK1, CETS1P54 |
| 7:23753048 | rs739403822 | G | A | CAAT, ZID, AHRARNT, COMP1, PADS, USF, AP2, SREBP1, EGR1, NGFIC, EGR3, EGR2 | ARP1, GATA2 |
| 7:23754967 | rs80692083 | T | C | S8, OCT, YY1, EVI1, SOX5 | MYB, VMYB, PAX5 |
| 7:23755992 | rs315864220 | C | T | OLF1, NGFIC, STAF, TAXCREB, LYF1 | EVI1, GATA1, GATA3, LMO2COM, OCT1, CHOP, BARBIE |
| 7:23756991 | rs314772558 | G | A | E2F, NRF2, LYF1 | POLY, ELK1, TAXCREB, GATA, OLF1, HSF2, AP1, GATA2, USF, DELTAEF1, SREBP1 |
| 7:23756996 | rs313942842 | A | G | | HOX13, COMP1, TATA, LYF1, SP1, MZF1, P300, GR |
| 8:2866334 | rs317104670 | C | T | CREBP1, HSF2, ARNT, USF, E2F | CAAT, AP4, EVI1, SRY |
| 10:7222733 | rs733428170 | T | A | CEBP | XFD3, CREB, GC, AP1, NFE2, GATA, P53, NRF2 |

（To be continued on next page）

`

（Continued from previous page）

| rs | rsID | WT | MT | Specific transcription factors binding before mutation | Specific transcription factors binding after mutation |
|---|---|---|---|---|---|
| 10:7224607 | rs80735721 | G | A | AP1FJ, GATA1, CAP | MYOGNF1, PBX1, SRY, RFX1, ISRE, VMYB, IRF1, IRF2 |
| 11:15476382 | rs15690110 | A | G | HFH1, ARP1 | CHOP |
| 11:15477314 | rs16519176 | C | T | OLF1, GATA1, GATA2, TAXCREB, HSF1, XBP1, CREBP1, CREB, SRF | OCT1 |
| 11:16237253 | rs731954564 | A | G | HOX13, S8, AP1, HNF1, OCT1, CDP, SOX5, VBP | NFY, SRY, BRACH |
| 11:16311558 | rs316739697 | G | A | MYCMAX | PAX6, BRN2, HOX13, AP1, GATA3, PBX1 |
| 11:16311635 | rs318127048 | G | C | BRN2, SRF, YY1, S8, PAX6, AHRARNT, P53, CREB | |
| 11:16323284 | rs314518447 | A | G | E2F, YY1, P53, HSF1, HSF2 | CEBP, CP2, OLF1, SOX5, ARP1 |
| 11:16348866 | rs14554663 | T | C | CEBPA, COUP, NFY, CEBP, AP1, CAP | OCT1, HSF1, NFKAPPAB65, CAAT, PBX1, STAF, CREBP1, GATA2, GATA3 |
| 12:1727650 | rs15743050 | C | G | STAF, VMAF, SP1, CEBPB, CEBP, USF, VBP, GATA1, GATA2, LMO2COM, VMYB | NRSF, NFY, ER, CAAT, CDPCR3HD |
| 12:11801847 | rs16527532 | A | G | CAAT, RFX1, XFD3, CEBPB, CEBP, IRF1, IRF2, VMYB, AP4, E2, NRF2 | BARBIE, STAF, E47, MZF1 |
| 12:11801904 | rs315866764 | T | C | TAXCREB, OCT1, AP1, HOX13, USF, PAX6, SREBP1, CLOX | NFKB, POLY, CETS1P54, GR, YY1, CDPCR1, CEBP |
| 12:11801963 | rs312861187 | G | A | COMP1 | MEF2, OCT1, XFD2, OCT, TH1E47, GATA1, HNF3B, GATA2, GATA3, S8, LMO2COM, EVI1, GATA, TATA, NKX25, ISRE, CEBPB |
| 12:11801979 | rs312491466 | A | G | CAAT | GR, POLY, ARNT, SOX5, CEBPB, CEBPA, HNF1, RFX1, TATA |

（To be continued on next page）

(Continued from previous page)

| rs | rsID | WT | MT | Specific transcription factors binding before mutation | Specific transcription factors binding after mutation |
|---|---|---|---|---|---|
| 12:11802112 | rs312592149 | T | A | GATA1, BRN2 | COMP1, HNF1, CEBP, CP2, EVI1, SOX5, E2 |
| 12:11802772 | rs312973670 | G | A | PAX5, MYOD, XBP1, USF | GATA3, GC, LMO2COM, GATA, CEBP, OCT1 |
| 12:11802862 | rs315588466 | T | C | E47, MYOD, BARBIE, RFX1, CP2 | P300, GC, SP1, IRF1, IRF2 |
| 12:11802865 | rs16530658 | T | C | ELK1, BARBIE, CP2 | E2, CETS1P54 |
| 12:11804201 | rs314184780 | A | G | OLF1, HEN1, TAL1BETAE47 | PAX5, R, SRF, EGR2, AP2, SP1, GC |
| 12:11804663 | rs15776234 | A | G | EVI1, XFD1, HNF3B, SOX5, BARBIE | ISRE, HSF2, LMO2COM, ELK1 |
| 12:11804863 | rs739635299 | C | A | ARP1, HOX13, T3R, HEN1, PADS, R, E47, LMO2COM, VMYB, NFY | CP2, E2F, YY1 |
| 12:19780775 | rs316811946 | C | T | SEF1, CREB, E2F, AHRARNT | AP4, MAX, USF, NMYC, DELTAEF1, SREBP1 |
| 12:19783140 | rs316188605 | A | C | IK1, MIF1, SRF, GATA1, CDP, IRF1, IRF2 | TAXCREB, STAF, MZF1, EGR2, CMYB |
| 12:19784461 | rs314985840 | A | G | CP2, NRF2 | GATA3, LMO2COM, GC |
| 12:19786586 | rs793931460 | C | T | R, HEN1, YY1, GRE, TAXCREB, CREB, TH1E47, AHRARNT | SREBP1, GATA1, GATA2, GATA3, LMO2COM, GATA, CLOX |
| 12:19801364 | rs739919594 | G | T | MYOGNF1, ARNT, AP2, CMYB, PAX5 | POLY, MAX, E2F, HEN1, ELK1, VMYB |
| 14:1238758 | rs316991790 | A | G | POLY, GC, VJUN, YY1, S8, NKX25, SRY, CEBP | VMYB, CREB |
| 14:2508316 | rs313792827 | C | A | HNF4, GFI1, PBX1, EVI1, GATA3 | HFH2, HNF3B, CDP, CAP, XFD2 |
| 14:14628939 | rs312457584 | C | A | AP4, CHOP, MYOGNF1, ZID | BRN2, PAX2, HSF2, COMP1, GATA2, GATA3, LMO2COM, CLOX, CDPCR1, CDPCR3HD, CEBP |
| 18:7916856 | rs313224745 | A | G | AHR, AHRARNT, XFD3, SRY, PAX2, CEBPB | CEBP, XBP1, ATF, RFX1 |
| 18:9947166 | rs740514701 | T | C | STAF, NFY | AP2, SP1 |
| 18:9950906 | rs316894769 | C | A | PAX2, TAXCREB, CP2 | AHR, E2F, IK3, CREB |

(To be continued on next page)

`

（Continued from previous page）

| rs | rsID | WT | MT | Specific transcription factors binding before mutation | Specific transcription factors binding after mutation |
|---|---|---|---|---|---|
| 18:9959147 | rs15864493 | C | G | GR, T3R, CDPCR3HD, S8, RORA1, RORA2, AP4 | XBP1, USF, TAXCREB, MYCMAX, SREBP1, CREB, CREBP1, CREBP1CJUN, HNF1, AP1FJ |
| 18:9960650 | rs314090469 | A | G | NFKAPPAB65, CREL, CEBP, OCT | GC, CP2, NRF2, CHOP, AHRARNT |
| 19:6505623 | rs14617318 | A | G | GC, RFX1, NGFIC, CDPCR1, CDPCR3HD | ARP1, E2, ARNT, NMYC, USF, NFKAPPAB65, CREL, HSF1, HSF2 |
| 22:461024 | rs14617319 | C | T | STAF, IRF2, COUP, MYCMAX, CMYB, ER, YY1, VMYB | E47, HNF1, HFH1, IK3, XBP1 |
| 27:3796945 | rs313296176 | G | A | COMP1, SRF, STAF, AP1 | TST1, PAX2, TATA, HNF1, BARBIE, SRY |
| Z:30960219 | rs313456709 | T | C | RREB1, XFD2, CEBP | COMP1 |
| Z:30982495 | rs730934738 | C | T | HNF1, VMYB | CDP, XFD1, GR, SRY, TST1 |
| Z:31467999 | rs312449117 | C | T | | STAF, YY1, BARBIE, CEBP, CEBPB, IK1, NF1, AHRARNT |
| Z:31468208 | rs738431855 | A | G | NFKB, GATA1, VMYB, MYB | ARP1, SP1, E47, LMO2COM, AP4, MYOD |
| Z:31468803 | rs338822069 4 | T | C | OCT1, SRY, TAL1BETAE47, TAL1BETAITF2, GATA1, GATA2, GATA3 | E47, MYOD, DELTAEF1, NRF2 |
| Z:31469397 | rs317018855 | C | T | CMYB, ARNT, ATF, HNF4, VMYB, CREBP1CJUN, PPARA, HSF2 | SREBP1, OCT1, HNF1, SOX5, XFD3, HFH1, HFH2, HNF3B |
| Z:31470828 | rs314490595 | T | C | HEN1, E47, MYOD, ZID, AP2 | COUP, AP4, CAP, NGFIC |
| Z:31471141 | rs313488012 | C | T | HSF1, HSF2, GATA3 | ARP1 |
| Z:31471295 | rs313387429 | T | C | HOX13, HSF1, HSF2, AP1, ARP1 | TAXCREB, NFKB, NFKAPPAB65, IK3, IK2, STAT, CDPCR1 |
| Z:31474541 | rs735129014 | T | G | GR, P300, CAP | PAX6, ARNT, AHRARNT, ARP1 |

（To be continued on next page）

(Continued from previous page)

| rs | rsID | WT | MT | Specific transcription factors binding before mutation | Specific transcription factors binding after mutation |
|---|---|---|---|---|---|
| Z:31474926 | rs733539751 | T | C | EVI1 | VMAF, CEBP, AP4, GR, GRE, VMYB |
| Z:31475239 | rs16776397 | T | C | CP2, CREB, RFX1, NF1 | AP1FJ, NFY |
| Z:31475464 | rs741291342 | A | T | RFX1, NFY, E2F, MYOGNF1, IK1, IK2, IK3, POLY, LYF1, NFKB, CREL, NFKAPPAB, OCT1 | |
| Z:31476489 | rs316071695 | G | T | YY1, S8, GATA1, E4BP4, GATA2, CREB, CREBP1, LYF1, STAT, IK3 | ARNT, MYCMAX, MAX, USF, RFX1, NMYC, CEBP, LMO2COM, MYOD, VMYB, CEBPB, CEBPA, BRN2, IK1, NFKB, CREL |
| Z:31478846 | rs733148472 | A | C | GC | GATA1, GATA2, STAT, LYF1 |
| Z:31481217 | rs736318169 | T | A | S8, P53, HFH1, HNF3B, CP2, PBX1 | CEBPB |
| Z:31492476 | rs314955625 | A | T | CEBPA, BRN2, E4BP4, VBP, HLF, CREBP1, CAP, NKX25 | S8, IRF1, XFD3 |
| Z:31492800 | rs313371449 | A | G | CLOX, PBX1, EVI1, SRY, HNF3B, NFY, CDP, CAAT, POLY | HOX13, CETS1P54, GATA1, GATA2, VMYB, LMO2COM |
| Z:31494181 | rs14778403 | A | G | RFX1, COMP1, CEBP, OCT, VMYB | AP2, TAL1BETAE47, TAL1BETAITF2, E47, AP4, MYOD, GATA1, GATA2, MYOGNF1, OLF1 |
| Z:31494359 | rs312440942 | T | C | XFD1, S8, MYOD, E2F | CAAT, GATA1, GATA2, GATA3, COMP1 |
| Z:31501598 | rs740312969 | T | C | STAT1, TH1E47, GC, GATA2, CAAT, CP2, MZF1 | COMP1, CEBP, GFI1, AP4 |
